# Imprinting methylation predicts hippocampal volumes and hyperintensities and the change with age in later life

**DOI:** 10.1038/s41598-020-78062-2

**Published:** 2021-01-13

**Authors:** Marlene Lorgen-Ritchie, Alison D. Murray, Roger Staff, Anne C. Ferguson-Smith, Marcus Richards, Graham W. Horgan, Louise H. Phillips, Gwen Hoad, Chris McNeil, Antonio Ribeiro, Paul Haggarty

**Affiliations:** 1 Rowett Institute of Nutrition and Health, University of Aberdeen, Aberdeen, AB25 2ZD UK; 2 Aberdeen Biomedical Imaging Centre, University of Aberdeen, Aberdeen, AB25 2ZD UK; 3 NHS Grampian, Aberdeen, UK; 4 Department of Genetics, University of Cambridge, Cambridge, CB2 3EH UK; 5 MRC Unit for Lifelong Health and Ageing at UCL, University College London, London, UK; 6 Biomathematics and Statistics Scotland, University of Aberdeen, Aberdeen, AB25 2ZD UK; 7 School of Psychology, University of Aberdeen, Aberdeen, AB24 3FX UK; 8 Centre for Genome-Enabled Biology and Medicine, University of Aberdeen, Aberdeen, AB24 3UU UK

**Keywords:** Imprinting, DNA methylation, Cognitive ageing

## Abstract

Epigenetic imprinting is important for neurogenesis and brain function. Hippocampal volumes and brain hyperintensities in late life have been associated with early life circumstances. Epigenetic imprinting may underpin these associations. Methylation was measured at 982 sites in 13 imprinted locations in blood samples from a longitudinal cohort by bisulphite amplicon sequencing. Hippocampal volumes and hyperintensities were determined at age 64y and 72y using MRI. Hyperintensities were determined in white matter, grey matter and infratentorial regions. Permutation methods were used to adjust for multiple testing. At 64y, *H19/IGF2* and *NESPAS* methylation predicted hippocampal volumes. *PEG3* predicted hyperintensities in hippocampal grey matter, and white matter. *GNASXL* predicted grey matter hyperintensities. Changes with age were predicted for hippocampal volume (*MEST1*, *KvDMR*, *L3MBTL*, *GNASXL*), white matter (*MEST1*, *PEG3*) and hippocampal grey matter hyperintensities (*MCTS2*, *GNASXL, NESPAS, L3MBTL, MCTS2, SNRPN*, *MEST1*). Including childhood cognitive ability, years in education, or socioeconomic status as additional explanatory variables in regression analyses did not change the overall findings. Imprinting methylation in multiple genes predicts brain structures, and their change over time. These findings are potentially relevant to the development of novel tests of brain structure and function across the life-course, strategies to improve cognitive outcomes, and our understanding of early influences on brain development and function.

## Introduction

Brain development in early life and across the life course is modulated by both genetic and environmental factors. Adverse childhood experiences are associated with adult brain structure^1,2^ and also with the presentation of adult psychiatric disorders and cognitive deficits^3^. Early life stress affects important neurodevelopmental processes, including neurogenesis, synaptic overproduction and pruning, and myelination during specific, sensitive periods^4^. The hippocampus in particular is known to be sensitive to early life stress^5^ and adult hippocampal volumes in late life have been associated with childhood socioeconomic status^6^. Childhood socioeconomic status was also associated with the prevalence of deep white matter and periventricular hyperintensities in a non-demented cohort in late mid-life^7^ and hyperintensities are associated with cognitive decline in adults^8^. The mechanisms underlying these effects are unknown but may act through foetal and/or early life programming.

Epigenetic imprinting^9^ is known to be important for neurogenesis, brain function and behaviour^10–14^ and a number of characteristics of imprinting make it particularly relevant to the study of early life effects. Imprints as a class are also generally stable over time, and for some imprints the original signal persists in a wide range of cell types many divisions and decades later^15,16^. This and the known sensitivity of some imprints to some aspects of the early environment^17–19^ suggests a potential mechanism through which the early environment may influence later biological structure and function. These characteristics also make imprints particularly amenable to study in longitudinal cohort designs where only blood samples may be available and it may not be possible to analyse tissues such as the brain.

We have previously reported on the link between selected imprints and cognitive ability^20^, but to our knowledge no previous studies have looked specifically at the role of epigenetic imprinting in brain integrity. In this study we hypothesize that associations exist between imprint methylation and hippocampal volume and the prevalence of brain MRI hyperintensities in the seventh decade of life in a well-characterised cohort born in 1936 and recruited at 64 years of age^21^. The potential role of childhood socioeconomic circumstance, cognitive ability, and years spent in education was assessed. The selected imprints studied were *NAP1L5, ZAC1, MEST1, H19/IGF2*, *IGF2*, *KvDMR*, *IGDMR*, *SNRPN*, *PEG3*, *MCTS2*, *L3MBTL*, *NESPAS* and *GNASXL*, all of which are primary imprinting control regions, with the exception of *IGF2* which is a secondary somatic imprint^22^. Methylation status was determined by next generation bisulphite amplicon sequencing covering 982 methylation sites.

We report results separately for deep brain white matter, periventricular white matter, grey matter and infratentorial hyperintensities as associations between brain hyperintensities and impaired cognition are dependent upon the location of lesions within the brain^23^.

## Results

The subject characteristics are shown in Table 1. The baseline sample consisted of 47.3% females and the follow-up of 49.4% females. All participants imaged at follow-up (age 72.8 ± 0.6) were also imaged at baseline (age 68 ± 0.7). Summary statistics for participant hippocampal volumes, total Scheltens’ scores and hyperintensities in different brain regions are shown in Table 2.

**Table 1:** Summary statistics for participant characteristics; weight (kg), height (cm), body mass index (BMI; kg/m^2^), adult socioeconomic status (Scottish Index of Multiple Deprivation decile; SIMD), number of years spent in education and childhood (Moray House Test (MHT) score) and adult (National Adult Reading Test (NART) score) cognitive ability.

Characteristic	Mean	SD	n
Weight (kg)	74.2	12.9	225
Height (cm)	166.3	8.7	225
BMI (kg/m^2^)	26.8	4.0	225
SIMD (decile)	7.0	3.0	231
Years in education	11.2	2.0	237
MHT score	45.0	10.6	236
NART score	110.5	8.9	235

**Table 2:** Summary statistics for participant hippocampal volume (HipV—mm^3^), total Scheltens’ score, number of deep white matter hyperintensities (WMH), number of periventricular white matter hyperintensities (PVWMH), number of grey matter hyperintensities (GMH), number of infratentorial hyperintensities (IFTH) and number of hippocampal grey matter intensities (HipGMH) at the time of MRI image collection. Only individuals with information on sex, total intracranial volume and age are included.

Variable	Baseline					Follow-up				
	Mean	SD	n	Median	IQR	Mean	SD	n	Median	IQR
Hippocampal Volume (mm^3^)	8116.4	840.7	237			7980.2	953.1	146		
Total Scheltens’ score	15.2	9.2	224			18.0	9.3	148		
WMH	7.2	4.8	224			8.2	4.7	148		
PVWMH	4.8	2.1	224			5.9	2.0	149		
GMH	2.2	2.6	224	1	3	2.6	3.1	149	1	4
IFTH	1.1	1.9	224	0	1	1.2	2.0	149	0	2
HipGMH	0.3	0.8	224	0	0	0.6	0.9	149	0	1

Medians and interquartile ranges (IQRs) are additionally reported for variables with non-normal distributions.

**Figure 1:**
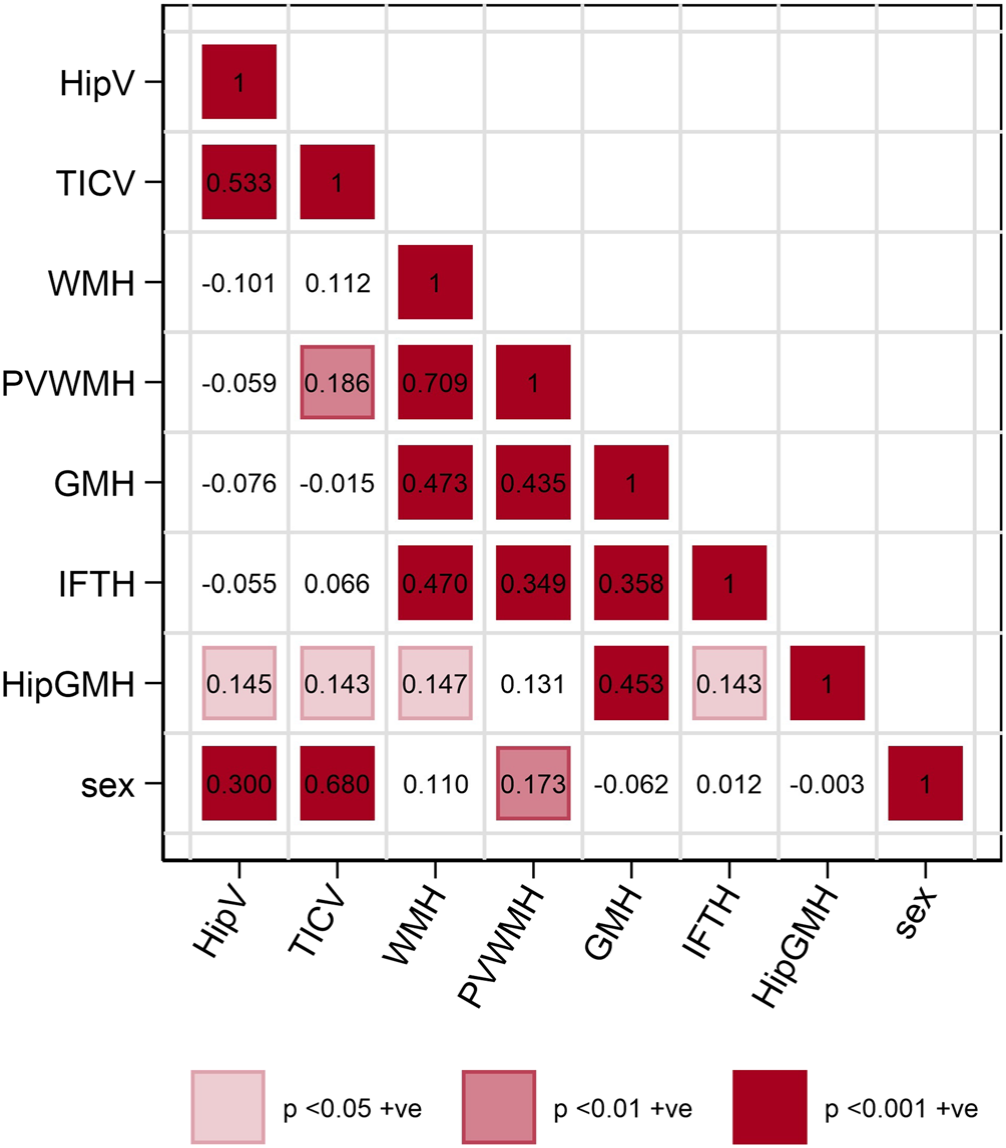
Correlation between hippocampal volume (HipV), total intracranial volume (TICV), number of deep white matter hyperintensities (WMH), number of periventricular white matter hyperintensities (PVWMH), number of grey matter hyperintensities (GMH), number of infratentorial hyperintensities (IFTH), number of hippocampal grey matter intensities (HipGMH) and sex at the time of baseline MRI image collection (age ~ 67–70). Colour gradients represent level of significance with most intense colour indicating the highest level of significance. Only individuals with information on sex, total intracranial volume and age are included. Created using STATA/MP version 15 (StataCorp. 2017. *Stata Statistical Software: Release 15*. College Station, TX: StataCorp LLC).

The correlation between outcome variables was assessed prior to the imprinting analysis (Fig. 1). Hippocampal volume was positively correlated with total intracranial volume and the number of grey matter hyperintensities specific to the hippocampus. Total intracranial volume was also positively corelated with the number of grey matter hyperintensities specific to the hippocampus, and with the number of periventricular white matter hyperintensities. The number of white matter hyperintensities was strongly positively correlated with the number of periventricular white matter hyperintensities, and also grey matter hyperintensities, infratentorial hyperintensities, and hippocampal grey matter hyperintensities. Periventricular white matter hyperintensities were also positively correlated with grey matter and infratentorial hyperintensities. Grey matter hyperintensities were positively correlated with infratentorial and hippocampal grey matter hyperintensities, and these variables were also positively correlated with each other. Sex (coded 0 for females, and 1 for males) was positively associated with hippocampal and total intracranial volume, and with the number of periventricular hyperintensities, i.e. these 3 variables were increased in males compared to females.

**Figure 2:**
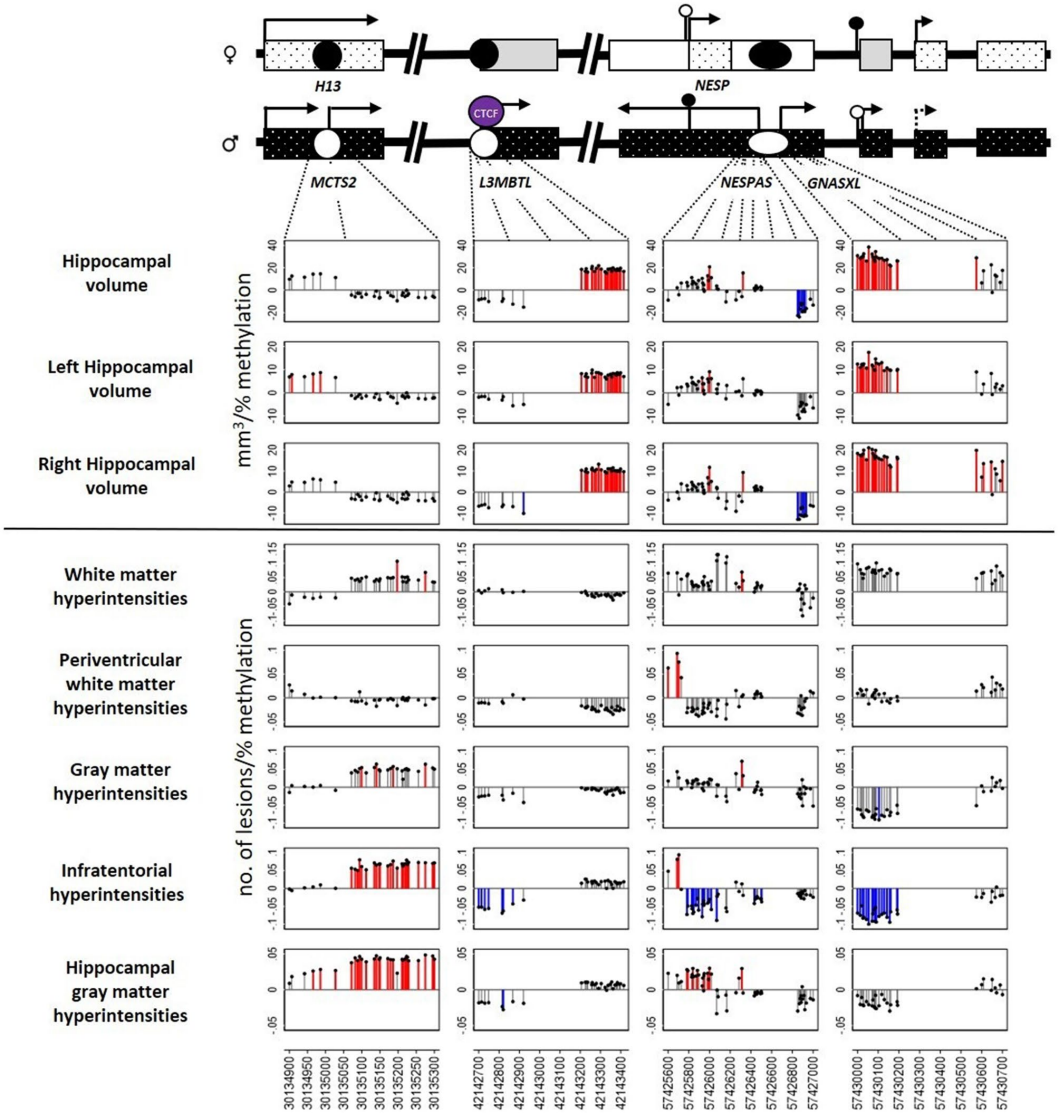
Example coefficient plot for associations between imprint methylation and change in hippocampal volumes and hyperintensities on chromosome 20 (*MCTS2*, *L3MBTL*, *NESPAS*, *GNASXL*) in ABC36 participants between baseline and follow-up image collection. The schematic at the top of each column of figures shows the genomic location of the individual CpG sites in the context of function on the maternally and paternally inherited alleles (indicated by male and female symbols). The y axes represent the change in hippocampal volume (in mm^3^) or the change in number of lesions per percent methylation and the x axes genomic location on chromosome 20 (GRCh37, release 84, version of March 2016). Positions significant at *p* < 0.05 are shown in blue when coefficients are negative and in red when positive. Regressions were adjusted for sex, total intracranial volume, age at baseline (in days) and time between MRI scans (in days). Created using STATA/MP version 15 (StataCorp. 2017. *Stata Statistical Software: Release 15*. College Station, TX: StataCorp LLC).

For the imprint analysis, linear regression was carried out for individual CpG positions against hippocampal volumes and Scheltens’ scores for different brain matter regions with adjustment for sex, total intracranial volume and age at baseline MRI. The analysis was repeated for the change in hippocampal volumes and Scheltens’ scores between baseline and follow-up with additional adjustment for time between MRI scans. The individual regression coefficients for each CpG were plotted consecutively to identify coherent blocks of significance. An example plot in which extensive significance was observed is presented in Fig. 2 for the methylation of imprints on chromosome 20 and the change in brain structure between scans. Equivalent figures for all the imprints and outcomes are in the supplementary material (Figures S1-S9). Statistically significant results are shown in red when the coefficient is of positive sign and blue when negative.

**Figure 3:**
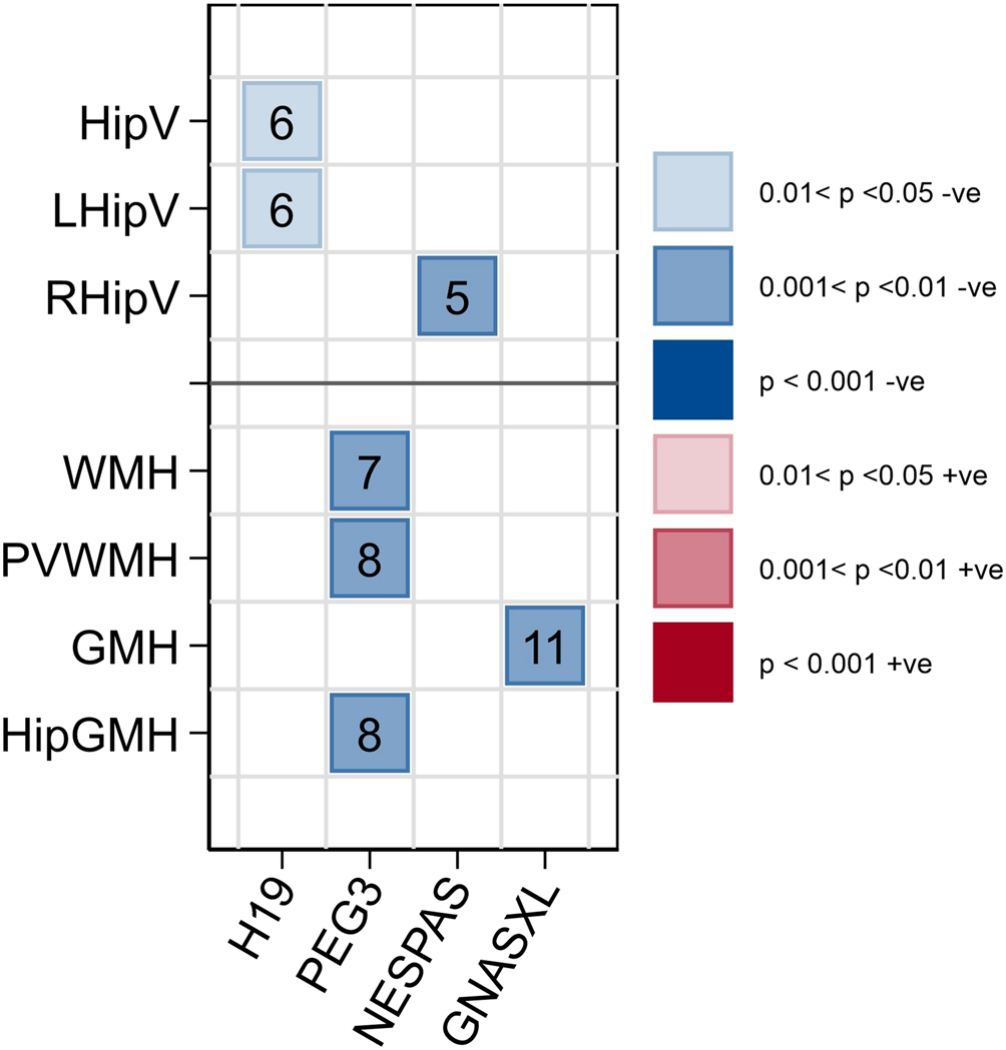
Heatmap showing significant blocks of association between imprint methylation and hippocampal volumes (in mm^3^) and hyperintensities at baseline MRI imaging following permutation analysis in ABC36 participants (HipV = hippocampal volume; LHipV = left hippocampal volume; RHipV = right hippocampal volume; WMH = white matter hyperintensities; PVWMH = periventricular hyperintensities; GMH = grey matter hyperintensities; HipGMH = grey matter hyperintensities specific to the hippocampus). Blue indicates negative associations and red positive. Colour gradients represent level of significance with most intense colour indicating the highest level of significance. Numbers of significant CpG sites in the association block are shown. Imprints are organised in chromosomal order along the x-axis. Created using STATA/MP version 15 (StataCorp. 2017. *Stata Statistical Software: Release 15*. College Station, TX: StataCorp LLC).

**Figure 4:**
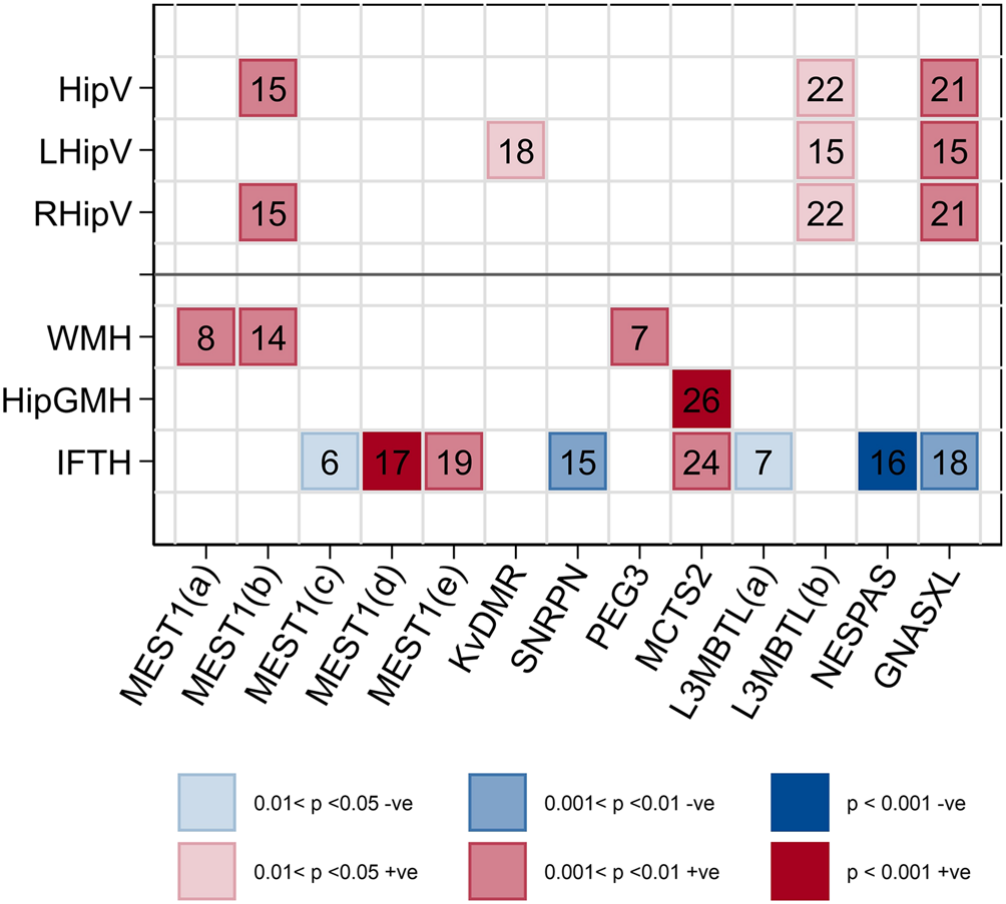
Heatmap showing significant blocks of association between imprint methylation and change in hippocampal volumes (in mm^3^) and hyperintensities between MRI imaging at baseline and follow-up in ABC36 participants following permutation analysis (HipV = hippocampal volume; LHipV = left hippocampal volume; RHipV = right hippocampal volume; WMH = white matter hyperintensities; HipGMH = grey matter hyperintensities specific to the hippocampus; IFTH = infratentorial hyperintensities). Blue indicates negative assoctions and red positive. Colour gradients represent level of significance with most intense colour indicating the highest level of significance. Numbers of significant CpG sites in the association block are shown. Imprints are organised in chromosomal order along the x-axis and vertically aligned squares cover the same CpG blocks. Created using STATA/MP version 15 (StataCorp. 2017. *Stata Statistical Software: Release 15*. College Station, TX: StataCorp LLC).

Permutation analysis was applied to groups of contiguous significance (see “Materials and methods”). The probability of the observed blocks of contiguous significance arising due to chance was determined from 1000 permutations of the methylation data. Summary graphical presentations of the data are shown in Figs. 3 and 4. Only outcomes for which at least one imprint was significant were included in the figures. Results which passed the threshold for statistical significance are shown in the heatmap type figure with positive correlations shown in red and negative in blue with the level of significance indicated by the colour intensity (see legend). Where there was more than one block of significance located in different regions of the same imprint these are shown separately. For each significant result represented in the figures the number of significant contiguous CpG sites is shown within the symbol.

Associations between methylation levels, and hippocampal volumes and hyperintensity prevalence at baseline MRI collection which met the significance criteria following permutation analysis are shown in Fig. 3. *H19/IGF2* methylation was negatively associated with hippocampal volume (− 26.5, *p* = 0.012), in particular the left hippocampus (− 13.3, *p* = 0.012) while *NESPAS* was negatively associated with right hippocampal volume (− 20.9, *p* = 0.001). *GNASXL* was negatively associated with the number of hyperintensities in grey matter (− 0.102, *p* = 0.004) while *PEG3* was negatively associated with the number of white matter hyperintensities (− 0.130, *p* = 0.005), periventricular white matter hyperintensities (− 0.070, *p* = 0.005) and the number of grey matter hyperintensities specific to the hippocampus (− 0.026, *p* = 0.005).

Significant associations with change in hippocampal volumes and hyperintensities between baseline and follow-up which met the significance criteria following permutation analysis are shown in Fig. 4. *MEST1* (+ 22.2, *p* = 0.007), *L3MBTL*(b) (+ 18.3, *p* = 0.018) and *GNASXL* (+ 28.7, *p* = 0.005) methylation were positively associated with change in total hippocampal volume. *MEST1* was exclusively associated with right hippocampal volume change (+ 14.1, *p* = 0.007) while *GNASXL* was associated with change in both right (+ 17.0, *p* = 0.005) and left (+ 12.4, *p* = 0.008) volumes. *L3MBTL*(b) was also associated with changes in both right (+ 10.5, *p* = 0.018) and left (+ 8.4, *p* = 0.002) hippocampal volumes while *KvDMR* methylation was positively associated with change in left hippocampal volume only (+ 10.3, *p* = 0.013). *MEST1* was also positively associated with a change in the number of white matter hyperintensities at two distinct regions (*MEST1*(a): + 0.131, *p* = 0.007; *MEST1*(b); + 0.112, *p* = 0.007). *PEG3* was also positively associated with change in white matter hyperintensity prevalence (+ 0.095, *p* = 0.004), and *MCTS2* with grey matter hyperintensities in the hippocampus (+ 0.041, *p* < 0.001). Change in the number of infratentorial hyperintensities was associated with a number of imprints, particularly those located on chromosome 20, where *NESPAS* showed a negative association (− 0.036, *p* < 0.001), as did *GNASXL* (− 0.079, *p* = 0.005) and *L3MBTL*(a) (− 0.056, *p* = 0.018), while *MCTS2* showed a positive association (+ 0.067, *p* = 0.004). *SNRPN* methylation showed a negative association (− 0.085, *p* = 0.004) and *MEST1* positive associations at 2 distinct regions (*MEST1*(d): + 0.097, *p* < 0.001; *MEST1*(e): + 0.090, *p* = 0.009), and a less significant negative association at a third block upstream (*MEST1*(c): − 0.096, *p* = 0.012).

*MEST1* associations with change in hippocampal volume and white matter hyperintensities occurred at the same genomic location, but the associations with infratentorial regions were in different regions of *MEST1*. *L3MBTL* associations with hippocampal volume and infratentorial hyperintensities also occurred in different regions within the imprint. *MCTS2* associations with change in hippocampal GMH and IFTH occurred in the same genomic location and were consistently positive. *GNASXL* was linked to hippocampal volumes and IFTH, although in the latter case the IFTH associations were in the opposite direction. Some of the associations covered a large number of contiguous CpGs (e.g. *MCTS2* and GMH and IFTH) and, in general, the higher the number of significant contiguous CpGs, the lower the likelihood of this arising by chance and greater the level of significance following permutation correction. Contiguous blocks of significance can also arise by chance because of correlation between adjacent CpGs. The permutation analysis specifically took account of the structure of the data and the autocorrelation between CpGs and this can be seen in cases where it reduced the level of significance despite there being a high number of contiguous CpGs (e.g. *L3MBTL* and hippocampal volumes).

No impacts upon patterns of significance were observed when regressions included childhood cognitive ability at age 11 or years spent in education or childhood or adult socioeconomic status as additional explanatory variables.

## Discussion

Hippocampal volumes and hyperintensities are influenced by both genetics^24,25^ and the environment^5^, and sometimes the effects of both have been reported in the same study^26^. Epigenetic mechanisms are relevant to both genetic and environmental effects as epigenetic states important to brain structure and function can be both modifiable by the environment and heritable, in their own right or as a result of genetic variation. One class of epigenetics—imprinting—is thought to be particularly important for neurogenesis, brain function and behaviour^11–14^. The fact that imprints are set early in development also makes this class of epigenetic signatures particularly relevant to the study of early life effects on brain development.

This study focused on imprinting methylation as a predictor of hippocampal volumes and hyperintensities and their change with age in later life. We used next generation sequencing to assess CpG methylation across multiple sites within important imprinted regions. There were numerous examples of single isolated CpGs being significantly related to brain structures but these were rejected on the basis of biological implausibility. We only report findings based on contiguous blocks of significance across multiple CpGs which survived permutation adjustment for multiple testing and correlation between adjacent sites.

The hippocampus plays an important role in learning and memory consolidation, affective behaviours, and mood regulation^27^ and hippocampal atrophy is accelerated in patients with Alzheimer’s disease^23,28,29^. We report that methylation within the imprinted genes, *H19/IGF2* and *NESPAS* predicted hippocampal volumes at baseline MRI measurement in the seventh decade of life. As with most studies of imprinting, the magnitude of methylation changes are modest^18,19,30–32^ but they have the potential to contribute to important biological effects^20^. Prenatal exposures resulting in a 1% change in methylation at the *IGF2* DMR correspond to an approximately twofold change in *IGF2* transcription^33^ and small changes in imprinting methylation also have the potential to influence the wider genome through short- and long-range interactions. Baseline hippocampal volumes in this cohort were found to differ by approximately 500 mm^3^ between individuals who went on to develop Alzheimer’s disease compared to those who did not^28^. A 1% change in *H19/IGF2* methylation predicted a 26 mm^3^ difference in total hippocampal volume and the population variation (two standard deviations) in methylation levels was 4%, equivalent to a fifth of the full Alzheimer’s effect. Methylation within *MEST1*, *KvDMR*, *L3MBTL* and *GNASXL* all predicted changes in hippocampal volume with age between baseline and follow-up. The hippocampus is known to be sensitive to early life stress^5^ and adult hippocampal volumes in late life have been associated with childhood socioeconomic status^6^. There is some evidence from human^34^ and animal studies^35^ that epigenetic change in non-imprinted genes may be relevant to the link between early life adversity and hippocampal development but the evidence for imprinted genes has so far been indirect or inferred. Offspring methylation within the imprinted gene *MEST* has been linked to exposure to maternal stress^36^, which has in turn been linked to hippocampal volumes, and there is some genetic evidence that the imprinted gene *L3MBTL* may work with a subnetwork of genes to influence hippocampal volume, microstructure and asymmetry^37^.

White matter hyperintensities in the brain are the consequence of cerebral small vessel disease and are important risk factors for cognitive and functional impairment dementia, stroke, worse outcomes after stroke, gait instability, late-life depression, and death^23,38,39^. There is also evidence for early life effects on these important indicators of function; particularly hyperintensities in white matter^7^. At baseline, *PEG3* methylation predicted the number of sub-cortical and deep white matter hyperintensities (frontal, temporal, parietal, occipital, internal capsule), periventricular white matter hyperintensities (frontal horns, bodies, occipital horns), and the number of grey matter hyperintensities specific to the hippocampus, while *GNASXL* methylation predicted total grey matter hyperintensities (caudate nucleus, putamen, globus pallidus, thalamus, hippocampus). Multiple imprints predicted the rate of change in hyperintensities with age between baseline and follow-up. Changes in white matter hyperintensities were predicted by *MEST1* and *PEG3* and changes in hippocampal grey matter hyperintensities by *MCTS2*. The number of imprints and regions predicting the rate of change in infratentorial hyperintensities (cerebellum, midbrain, pons, medulla) was striking, covering methylation within *GNASXL, NESPAS, L3MBTL, MCTS2, SNRPN*, and *MEST1* which showed significance in three different regions. The concentration of this effect in imprints within chromosome 20 was notable. All three imprints at the 20q13 locus (*L3MBTL*: q13.2; *GNASXL*: q13.32 and *NESPAS*: q13.32) demonstrated reduced methylation with increasing prevalence of hyperintensities and *MCTS2* – also on chromosome 20 but at location 20q11.21—showed a positive association.

This is the first study looking specifically at the role of epigenetic imprinting in brain hyperintensities and hippocampal volumes but, as with hippocampal volumes, there is some indirect or inferred evidence pointing to a possible role for imprints. *H19* expression is important in neuronal apoptosis and glial cell activation in the hippocampus in animal models^40,41^. Hyperintensities are associated with cognitive impairment and the *GNAS* locus has been linked to cognitive impairment syndromes^42,43^, while *MEST1* and *SNRPN* predict cognitive ability in childhood^20^. *SNRPN* has also been associated with dendritic spine development during postnatal brain development^44,45^. The neurodegenerative disease of multiple sclerosis is characterised by hyperintensities in the infratentorial region^46^ and a susceptibility locus for multiple sclerosis has been identified at 20q13 which contains *L3MBTL* and *GNAS*^47^.

We focused on imprints as a class because of their known role in brain development and function and in some cases common patterns emerge, such as the consistent direction of all the effects on hyperintensities and hippocampal volumes at baseline and the generally opposite direction of effect on the changes with age. There are also examples of individual imprints affecting more than one outcome, such as *PEG3* predicting three of the four measures of hyperintensity. However, while imprints can be grouped together as a class of epigenetic phenomena, individual imprints have their own specific roles and functions, as discussed above, and the processes that lead to the establishment of a biological structure or function and those that determine its response to ageing are not necessarily the same. Even within a structure such as the hippocampus different processes may be active in different regions; e.g. asymmetric hippocampal volumes^29^ and atrophy have been identified with ageing^48^ and with increasing severity of Alzheimer’s disease^49^. It should also be noted that the significant findings are those that survived the application of a rigorous permutation test and where for example there were similar directions of effect in both sides of the hippocampus, only the findings on one side may have met the stringent level of significance imposed by the permutation analysis. In some imprints multiple regions of significance were observed; for example in *MEST1* where four regions were significantly linked to the same direction of ageing effect in the hippocampus and two measures of hyperintensity. Even within an imprint there are regions that cover different transcription factor binding sites and where methylation at different sites has different effects (e.g. the *H19/IGF2* locus in mouse models^50,51^) and a fifth region in *MEST1* was oppositely linked to one of the measures of hyperintensity.

We present evidence linking hippocampal volumes and hyperintensities, and their change over time, to contiguous extensive blocks of methylation within multiple imprints. Epigenetic imprinting in general is known to be important for neurogenesis, brain function and behaviour^11,12,14^ and we have identified specific imprints, and specific locations within those imprints, where contiguous blocks of methylation predict brain structure and its change over time. Shared mechanisms, driven by genomic imprinting, have the potential to connect early life exposures with brain structure and function and cognitive decline in old age.

Some strengths and limitations of the current study should be noted. The ABC36 longitudinal cohort is well-characterised and includes non-demented, community dwelling participants from a small geographical area who were born in same year, 1936. The longitudinal nature of the cohort study provides invaluable MRI data from single individuals across time in old age. As with many cohort studies, participants who underwent brain MRI were healthier and had higher cognitive ability than the cohort as a whole and it excludes anyone who died before recruitment age 64 years^28^.

Genetic effects can also influence epigenetic status through variation in the underlying genomic sequence of the region being measured^52^, or in proximal elements^53^, or by influencing the epigenetic machinery required for setting and maintaining imprints. An effect of the underlying genetic sequence within the imprinted regions studied here can be ruled out as an explanation for the findings as SNPs were specifically excluded from the regions analysed. It is possible that genetic effects operating at a distance could influence the relationships but the imprint specific direction of effect is not consistent with genetic variation within the general epigenetic read–write machinery. The significant results presented here were unaffected by inclusion of childhood or adult socioeconomic status, childhood cognitive ability, or the number of years spent in education as additional explanatory factors in regression analyses. This suggests that the links to function are biological rather than a consequence of indirect effects of imprints on behaviour and environmental exposures which then influence hippocampal volumes and hyperintensities.

Hippocampal volumes and hyperintensities are important corollaries of brain function and cognitive ability and an improved understanding of the factors that shape them would help improve the brain health of a substantial portion of older populations and reduce cognitive decline and Alzheimer disease. Innovative MRI studies have provided important advances in this field^39^. Epigenetic status within the imprinted genes provides a new front in our understanding of how the early environment can influence brain structure and function and new knowledge on which to develop novel strategies to improve brain health.

## Methods

### Cohort study design

Participants were members of the Aberdeen Birth Cohort of 1936 (ABC1936) from whom DNA was collected (n = 485)^21^. Ethical approval for the study was obtained from the Multi-Centre Research Ethics Committee for Scotland (MREC/01/0/56) and Grampian Research Ethics Committee (LREC/01/0299). The research was conducted in compliance with the Helsinki Declaration and all participants gave written, informed consent.

Almost all children born in 1936 and attending school in Scotland were tested at age 11 (± 0.5) years for general cognitive ability^54^. The test administered was a version of the Moray House Test No. 12, which was concurrently validated against the Terman-Merrill revision of the Binet Scales with a coefficient of approximately 0.8. Aberdeen survivors were traced and recruited to the study at mean age 64 years. Participants also gave blood samples at this age, and weight and height were measured by trained research nurses. In 2004, a target number of 250 members of ABC36, selected at random, were invited to undergo brain MRI. 249 participants agreed and were scanned at a mean age 68.6 years. 166 of participants agreed to a follow-up MRI scan at a mean age of 72.8 years. 149 participants were able to comply with imaging procedures and have MRI scans at both waves of collection^7,23,28^.

As described by Murray et al.^23^, participants who agreed were imaged using a 1.5 T GE CVi/NVi MR scanner (General Electric Medical Systems, Milwaukee, WI) between 2003 and 2005 using T2 axial (TR/TE 4900/81.4, slice thickness 5 mm, space 1.2 mm), fluid attenuation inversion recovery axial (FLAIR) (TR/TE 9002/1.33, TI 220, slice thickness 5 m mm, space 1.2 mm) and 3D T1 weighted spoiled gradient recalled acquisition (SPGR) (TR/TE 20/6 ms, flip angle 35°, number of slices 124, effective slice thickness 1.6 mm, matrix 256 × 192, in-plane resolution 1 × 1 mm) sequences. Complete T2 and FLAIR MRI data were available in 243 and completed 3DT1 data in 233, the smaller number being due to movement during the relatively longer volumetric acquisition. 3DT1 images were analysed using FreeSurfer to extract total intracranial, whole brain and hippocampal volumes (http://surfer.nmr.mgh.harvard.edu/). T2 and FLAIR images were analysed suing Scheltens’ scale^55^ which attributes scores based on hyperintensity size and number in the sub-cortical and deep white matter (WMH; fontal, temporal, parietal, occipital, internal capsule), periventricular white matter (PVH; frontal horns, bodies, occipital horns), deep grey matter (GMH; caudate nucleus, putamen, globus pallidus, thalamus, hippocampus) and infratentorial regions (IFTH; cerebellum, midbrain, pons, medulla).

### DNA extraction and BIS conversion

DNA extraction was carried out as described in^56^. DNA was extracted from blood samples (n = 485) using QIAamp DNA Blood Mini QIAcube kits (Qiagen, Crawley, UK), automated on a QIAcube (Qiagen, Crawley, UK). The DNA was quantified with SYBR Green on a Rotor-Gene Q (Qiagen, Crawley, UK) using DNA standards (Life Technologies, Paisley, UK). The DNA samples were dispensed into 96-well plates and treated with sodium bisulphite using Zymo EZ DNA Methylation-Gold Bisulphite kits (Zymo Research, California, USA) as described elsewhere^20^.

### Assay design

Germline DMRs were identified as those consistently identified as such across a number of publications^16,22,57–59^ (Table S1). FASTA sequences were used to design specific assays using PyroMark Assay Design Software (version 2.0, Qiagen, Crawley, UK). The optimum target size for all assays was 250 bp along with a Tm difference ≤ 2 °C between forward and reverse primers. CpG positions were avoided where possible in assay design, but where they could not be, inclusion was directed toward the 5′ end of the primer as a degenerate Y (or R for reverse primers) base. Assays were designed to avoid sequence variants in the primers. Primers sequences are shown in Table S2.

### PCR amplification

PCR products were generated using ZymoTaq DNA polymerase kits (Zymo Research) according to the manufacturer’s protocols. Forward and reverse primers were used at a final concentration of 0.75 µM each with 10 ng of bisulphite treated DNA in a final volume of 25 µl. Thermocycling conditions were as follows: 95 °C for 15 min, followed by 50 cycles of 95 °C for 30 s, annealing temperature (see Table S2) for 30 s, and 72 °C for 30 s, and a final extension for 7 min at 72 °C.

### Library preparation

For each individual, 2 µL of PCR product from each assay was added to an amplicon pool. Amplicon pools were quantified using the Thermo Fisher Scientific Quant-iT dsDNA High Sensitivity Assay (Thermo Fisher Scientific, Waltham, MA, USA). Fluorescence was measured using a BMG Labtech FLUOstar Omega microplate reader (BMG Labtech GmbH, Ortenberg, Germany). The amplicon pools were prepared for sequencing and barcoded using the NEB Ultra II DNA Library Prep Kit for Illumina (New England Biolabs, Ipswich, MA, USA) and the NEBNext Multiplex Oglios for Illumina (Dual Index Primers Set 1 and 2). The prepared libraries were quantified using the Thermo Fisher Scientific Quant-iT dsDNA High Sensitivity Assay with fluorescence measured on a BMG Labtech FLUOstar Omega microplate reader. The libraries were analysed for quality and size on an Agilent 2200 TapeStation with the High Sensitivity D1000 ScreenTape. Equimolar pooling of the resultant barcoded libraries was conducted and qPCR based quantification using the KAPA Complete Kit for Illumina Library Quantification (Roche Diagnostics, Risch-Rotkeuz, Switzerland) was completed on a Thermo Fisher Scientific QuantStudio 6 Flex Real-Time PCR System. The final library pools were sequenced on an Illumina MiSeq System employing v3 MiSeq chemistry and 300 bp paired-end reads. Base calls and FASTQ output files were produced on the MiSeq instrument. Two flow cells were run due to barcode number restraints.

### Quality control of sequencing data

Assessment of the number of sequencing reads and quality of the data were made for all samples using FastQC (version 0.11.5)^60^ and MultiQC (version 0.9.dev0)^61^ using default parameters. Raw reads from each of the samples were filtered to remove poor quality sequences and stringently trimmed to remove contaminating adapter sequences as well as any unwanted bias from their ends using Trim Galore! (version 0.4.0)^62^ as well as Trimmomatic (version 0.35)^63^. A phred score of 30 was used as the overall quality threshold. Custom scripts developed by Centre for Genome-Enabled Biology and Medicine’s (CGEBM’s) specialist Dr Antonio Ribeiro were utilised for the automation/parallelisation of the different operations across all the samples.

### Methylation calling

Methylation calling was carried out following the Bismark Bisulfite Mapper workflow^64^. In order to automate/parallelise the execution of the distinct stages of the workflow across all the samples, additional custom scripts were again developed by CGEBM’s Dr Antonio Ribeiro. For the mapping of the paired-end reads stage, Bowtie2^65^ as well as SAMtools version 0.1.19^66,67^ were employed over a mock genome built to comprise the targeted regions described in Table S1. The mock genome was based on the human genome build GRCh37, release 84, version of March 2016. A sub-set of samples were also aligned against the whole genome as verification of the mock genome approach. Additional Bismark-related parameters were set up as –un --ambiguous --non_bs_mm --non_directional –I 0 –X 1000, following recommendations from Bismark’s developer Felix Krueger (via personal communication). Due to the nature of the samples/library preparation, the deduplication of reads step was not performed. In the subsequent methylation extraction stage, Bismark and SAMtools were employed with paired-end-related parameters –p --no_overlap as well full reporting associated ones --report --no_header --bedGraph --zero_based --CX --buffer_size 50% --scaffolds --cytosine_report --CX --zero_based.

### Methylation reporting

Since Bismark reporting output format did not fulfil our requirements, a set of bespoke scripts were developed by CGEBM’s Dr Antonio Ribeiro to produce output formats more appropriate for subsequent downstream analysis. In this set of scripts, one component was responsible for the automation of the process across all samples while the other handled the formatting task. Specifically, the latter combines the information available in Bismark’s methylation extractor output files for coverage as well as for cytosines per context and per possible strand with the sequences of the targeted regions (in FASTA format) plus corresponding VCF files. As outputs, two distinct types of tabulated reports are generated: one detailing the methylation calling occurrences, with per-base resolution, and, the other breaking down the sequencing reads which supported such events. For the preparation of the aforementioned VCF files, Tabix tool version 0.2.5 (r964)^68^ was applied over the ENSEMBL’s “Homo_sapiens.vcf.gz” genetic variation information file^69^ respective to the human genome build GRCh37, release 84, version of March 2016.

### Statistical analyses

Statistical analysis was carried out using STATA/MP version 15 (Stata Corp, College Station, Texas, USA). Kernel density plots were drawn for each individual CpG position to identify any unusual methylation distributions that could be explained by nearby SNPs. These positions (n = 46 out of 982 CpGs) were excluded from further analyses. CpG methylation values were included in analyses only when there was at least 250 × cover on each strand (original top (OT) and complementary to original top (CTOT) or original bottom (OB) and complementary to original bottom (CTOB)). Methylation values less than 10% or greater than 90% were rare (< 1% of total) and sporadic but where they occurred they were excluded as unreliable or indicative of loss of imprinting. Mean methylation at each DMR is reported in Table S3.

Linear regressions were carried out for baseline and follow-up MRI at individual CpG positions against hippocampal volumes (mm^3^) and Scheltens’ scores (counts) for different brain matter regions with adjustment for sex, total intracranial volume and age at baseline MRI collection (in days). The analysis was repeated for the change in hippocampal volumes and Scheltens’ scores between baseline and follow-up MRI scans with additional adjustment for time between scans (in days). MRI variables were normally distributed, with the exceptions of total grey matter, hippocampal grey matter and infratentorial hyperintensities at baseline which were highly skewed toward scores of 0. Poisson regression with robust standard errors was additionally carried out for these variables to confirm positive results from linear regression and showed highly similar patterns of association with stronger *p*-values. Methylation was the explanatory variable in all regressions. The role of MHT score at age 11, the number of years spent in education, adult (aSES) and childhood socioeconomic status (cSES) in understanding these relationships was assessed by including these variables individually in further analyses. The Scottish Index of Multiple Deprivation (SIMD) decile was used as the measure of socioeconomic circumstances at the time of blood sampling. cSES was calculated as the standardized first component derived by PCA using overcrowding variables (sanitation sharing and room sharing) and also father’s occupation (coded 1 to 9 where 1 is higher managerial and 9 is never worked or long term unemployed). To maintain the number of individuals in these adjusted regressions, missing values were imputed using means. The results of all regressions were visualised in spike plots showing the magnitude, direction of association, and occurrence of statistical significance for each imprint in relation to the physical genomic location of the CpG. Significance at the *p* < 0.05 level was shown in red for positive associations and blue for negative associations. Regression results were plotted only when a minimum of n = 100 individuals were present in the regression analysis at an individual position.

### Permutation analysis

Regression analysis of multiple individual CpG sites increases the likelihood of significance arising by chance and, even if individual sites are truly statistically significant, the biological interpretation of such isolated CpG signals is not clear. Therefore, the focus here was in identifying blocks of significance covering multiple contiguous CpGs. Potential blocks of significance were identified for groups of adjacent significant CpGs with no more than one non-significant site between them. We used permutation analysis to assess the likelihood of important blocks of significance occurring by chance and the likelihood is reported in the results section. Adjustment for multiple testing in this case is complicated by the fact that there may be correlation in the methylation level between adjacent CpG sites. The autocorrelation structure was maintained in the permutation analysis by retaining the original methylation signal and regressing each CpG against a randomly generated normally distributed data set. This process was repeated 1000 times. Mean coefficients were derived by taking the mean across coefficients at individually significant CpGs within identified blocks of significance.

## Supplementary information

Supplementary Information.

## Data Availability

Applications to access the study data should be made to the Aberdeen Birth Cohort steering committee (c.mcneil@abdn.ac.uk).

## References

[1] Vythilingam M (2002). Childhood trauma associated with smaller hippocampal volume in women with major depression. Am. J. Psychiatry.

[2] Bremner JD (2003). Long-term effects of childhood abuse on brain and neurobiology. Child Adolesc. Psychiatr. Clin. N. Am..

[3] Hedges DW, Woon FL (2011). Early-life stress and cognitive outcome. Psychopharmacology.

[4] Teicher MH (2003). The neurobiological consequences of early stress and childhood maltreatment. Neurosci. Biobehav. Rev. Brain Dev Sex Differ. Stress Implic. Psychopathol..

[5] Gould E, Tanapat P (1999). Stress and hippocampal neurogenesis. Biol. Psychiatry.

[6] Staff RT (2012). Childhood socioeconomic status and adult brain size: childhood socioeconomic status influences adult hippocampal size. Ann. Neurol..

[7] Murray AD, McNeil CJ, Salarirad S, Whalley LJ, Staff RT (2014). Early life socioeconomic circumstance and late life brain hyperintensities: a population based cohort study. PLoS ONE.

[8] Murray AD (2012). Brain lesions, hypertension and cognitive ageing in the 1921 and 1936 Aberdeen birth cohorts. Age (Dordrecht, Netherlands).

[9] Ferguson-Smith A (2011). Genomic imprinting: the emergence of an epigenetic paradigm. Nat. Rev. Genet..

[10] Levenson JM, Sweatt JD (2005). Epigenetic mechanisms in memory formation. Nat. Rev. Neurosci..

[11] Wilkinson LS, Davies W, Isles AR (2007). Genomic imprinting effects on brain development and function. Nat. Rev. Neurosci..

[12] Badcock C (2011). The imprinted brain: how genes set the balance between autism and psychosis. Epigenomics.

[13] Ferrón SR (2011). Postnatal loss of Dlk1 imprinting in stem cells and niche astrocytes regulates neurogenesis. Nature.

[14] Kopsida E, Mikaelsson MA, Davies W (2011). The role of imprinted genes in mediating susceptibility to neuropsychiatric disorders. Horm. Behav..

[15] Coolen MW (2011). Impact of the genome on the epigenome is manifested in DNA methylation patterns of imprinted regions in monozygotic and dizygotic twins. PLoS ONE.

[16] Woodfine K, Huddleston JE, Murrell A (2011). Quantitative analysis of DNA methylation at all human imprinted regions reveals preservation of epigenetic stability in adult somatic tissue. Epigenet. Chromatin..

[17] Haggarty P, Ferguson-Smith A, Kuh D, Cooper R, Hardy R, Richards M, Ben-Shlomo Y (2013). A Life Course Approach to Healthy Ageing.

[18] Haggarty P (2013). Folate in pregnancy and imprinted gene and repeat element methylation in the offspring. Am. J. Clin. Nutr..

[19] Whitelaw N (2014). Epigenetic status in the offspring of spontaneous and assisted conception. Hum. Reprod..

[20] Lorgen-Ritchie M (2019). Imprinting methylation in SNRPN and MEST1 in adult blood predicts cognitive ability. PLoS ONE.

[21] Whalley LJ (2011). How the 1932 and 1947 mental surveys of Aberdeen schoolchildren provide a framework to explore the childhood origins of late onset disease and disability. Maturitas.

[22] Hanna CW (2016). Pervasive polymorphic imprinted methylation in the human placenta. Genome Res..

[23] Murray A (2016). Brain hyperintensity location determines outcome in the triad of impaired cognition, physical health and depressive symptoms: a cohort study in late life. Arch. Gerontol. Geriatr..

[24] Sachdev Perminder S (2016). White matter hyperintensities are under strong genetic influence. Stroke.

[25] Mather KA (2015). Investigating the genetics of hippocampal volume in older adults without dementia. PLoS ONE.

[26] van Erp TGM (2002). Contributions of genetic risk and fetal hypoxia to hippocampal volume in patients with schizophrenia or schizoaffective disorder, their unaffected siblings, and healthy unrelated volunteers. Am. J. Psychiatry.

[27] Bettio LEB, Rajendran L, Gil-Mohapel J (2017). The effects of aging in the hippocampus and cognitive decline. Neurosci. Biobehav. Rev..

[28] Rana AK (2016). A comparison of measurement methods of hippocampal atrophy rate for predicting Alzheimer's dementia in the Aberdeen Birth Cohort of 1936. Alzheimer's Dement. (Amsterdam, Netherlands).

[29] Nobis L (2019). Hippocampal volume across age: Nomograms derived from over 19,700 people in UK Biobank. NeuroImage Clin..

[30] Soubry A (2016). Obesity-related DNA methylation at imprinted genes in human sperm: results from the TIEGER study. Clin. Epigenet..

[31] Soubry A (2015). Newborns of obese parents have altered DNA methylation patterns at imprinted genes. Int. J. Obes..

[32] Heijmans BT (2008). Persistent epigenetic differences associated with prenatal exposure to famine in humans. Proc. Natl. Acad. Sci. USA.

[33] Murphy SK (2012). Gender-specific methylation differences in relation to prenatal exposure to cigarette smoke. Gene.

[34] McGowan P (2009). Epigenetic regulation of the glucocorticoid receptor in human brain associates with childhood abuse. Nat. Neurosci..

[35] Meaney MJ, Szyf M (2005). Environmental programming of stress responses through DNA methylation: life at the interface between a dynamic environment and a fixed genome. Dial. Clin. Neurosci..

[36] Hollis F, Wang H, Dietz D, Gunjan A, Kabbaj M (2010). The effects of repeated social defeat on long-term depressive-like behavior and short-term histone modifications in the hippocampus in male Sprague–Dawley rats. Psychopharmacology.

[37] Ong M (2019). Neonatal amygdalae and hippocampi are influenced by genotype and prenatal environment, and reflected in the neonatal DNA methylome. Genes Brain Behav..

[38] Prins ND, Scheltens P (2015). White matter hyperintensities, cognitive impairment and dementia: an update. Nat. Rev. Neurol..

[39] de Havenon A, Meyer C, McNally JS, Alexander M, Chung L (2019). Subclinical cerebrovascular disease: epidemiology and treatment. Curr. Atheroscler. Rep..

[40] Han C (2018). Long non-coding RNA H19 contributes to apoptosis of hippocampal neurons by inhibiting let-7b in a rat model of temporal lobe epilepsy. Cell Death Dis..

[41] Han C (2018). LncRNA H19 contributes to hippocampal glial cell activation via JAK/STAT signaling in a rat model of temporal lobe epilepsy. J. Neuroinflamm..

[42] Butler MG (2009). Genomic imprinting disorders in humans: a mini-review. J. Assist. Reprod. Genet..

[43] Albright F, Burnett C, Smith P, Parson W (1942). Pseudo-hypoparathyroidism—an example of 'Seabright-Bantam syndrome': report of three cases. Endocrinology.

[44] Li H (2016). The autism-related gene SNRPN regulates cortical and spine development via controlling nuclear receptor Nr4a1. Sci. Rep..

[45] Kasai H, Fukuda M, Watanabe S, Hayashi-Takagi A, Noguchi J (2010). Structural dynamics of dendritic spines in memory and cognition. Trends Neurosci..

[46] Oksenberg JR, Barcellos LF (2005). Multiple sclerosis genetics: leaving no stone unturned. Genes Immun..

[47] The Australia and New Zealand Multiple Sclerosis, Genetics Consortium (2009). Genome-wide association study identifies new multiple sclerosis susceptibility loci on chromosomes 12 and 20. Nat. Genet..

[48] Ystad MA (2009). Hippocampal volumes are important predictors for memory function in elderly women. BMC Med. Imaging.

[49] Sarica A (2018). MRI asymmetry index of hippocampal subfields increases through the continuum from the mild cognitive impairment to the alzheimer's disease. Front. Neurosci..

[50] Phillips JE, Corces VG (2009). CTCF: master weaver of the genome. Cell.

[51] Murrell A (2001). An intragenic methylated region in the imprinted Igf2 gene augments transcription. EMBO Rep..

[52] Schubeler D (2015). Function and information content of DNA methylation. Nature.

[53] Lienert F (2011). Identification of genetic elements that autonomously determine DNA methylation states. Nat. Genet..

[54] Penrose LS (1949). The Trend of Scottish Intelligence: A Comparison of the 1947 and 1932 Surveys of the Intelligence of Eleven-Year-Old Pupils xxviii, 151-xxviii, 151.

[55] Scheltens P (1993). A semiquantative rating scale for the assessment of signal hyperintensities on magnetic resonance imaging. J. Neurol. Sci..

[56] Harrison K (2015). Breast cancer risk and imprinting methylation in blood. Clin. Epigenet..

[57] Das R (2013). DNMT1 and AIM1 imprinting in human placenta revealed through a genome-wide screen for allele-specific DNA methylation. BMC Genom..

[58] Pervjakova N (2016). Imprinted genes and imprinting control regions show predominant intermediate methylation in adult somatic tissues. Epigenomics.

[59] Court F (2014). Genome-wide parent-of-origin DNA methylation analysis reveals the intricacies of human imprinting and suggests a germline methylation-independent mechanism of establishment. Genome Res..

[60] http://www.bioinformatics.babraham.ac.uk/projects/fastqc.

[61] Ewels P, Magnusson M, Lundin S, Käller M (2016). MultiQC: summarize analysis results for multiple tools and samples in a single report. Bioinformatics.

[62] http://www.bioinformatics.babraham.ac.uk/projects/trim_galore/.

[63] Bolger AM, Lohse M, Usadel B (2014). Trimmomatic: a flexible trimmer for Illumina sequence data. Bioinformatics.

[64] Krueger F, Andrews SR (2011). Bismark: a flexible aligner and methylation caller for Bisulfite-Seq applications. Bioinformatics.

[65] Langmead B, Salzberg SL (2012). Fast gapped-read alignment with Bowtie 2. Nat. Methods.

[66] Li H (2009). The sequence alignment/map format and SAMtools. Bioinformatics.

[67] Li H (2011). Improving SNP discovery by base alignment quality. Bioinformatics.

[68] Li H (2011). Tabix: fast retrieval of sequence features from generic TAB-delimited files. Bioinformatics.

[69] ENSEMBLE. Index of /pub/grch37/release-84/variation/vcf/homo_sapiens. 17 November. Accessed 16 Feb 2016. http://ftp.ensembl.org/pub/grch37/release-84/variation/vcf/homo_sapiens/.

