# Supplementary material for: Imprinting methylation predicts hippocampal volumes and hyperintensities and the change with age in later life
Source: Sci Rep. 2021 Jan 13;11:943. doi: 10.1038/s41598-020-78062-2 (PMC7806645; doi:10.1038/s41598-020-78062-2)
Supplement: Supplementary file 1 — Supplementary Information. [file 41598_2020_78062_MOESM1_ESM.pdf]

Imprinting methylation predicts hippocampal volumes and hyperintensities and the change with age in later life.

Supplemental material.

Marlene Lorgen-Ritchie[1], Alison D. Murray[2], Roger Staff[3], Anne C. Ferguson-Smith[4], Marcus Richards[5], Graham W. Horgan[6], Louise H. Phillips[7], Gwen Hoad[1], Chris McNeil[2], Antonio Ribeiro[8], Paul Haggarty[1*]

[1] Rowett Institute of Nutrition and Health, University of Aberdeen, Aberdeen, AB25 2ZD, UK.

[2] Aberdeen Biomedical Imaging Centre, University of Aberdeen, Aberdeen, AB25 2ZD UK.

[3] NHS Grampian, Aberdeen, UK

[4] Department of Genetics, University of Cambridge, Cambridge, CB2 3EH, UK

[5] MRC Unit for Lifelong Health and Ageing at UCL, University College London, UK

[6] Biomathematics and Statistics Scotland, University of Aberdeen, Aberdeen, AB25 2ZD, UK.

[7] School of Psychology, University of Aberdeen, AB24 3FX, UK

[8] Centre for Genome-Enabled Biology and Medicine, University of Aberdeen, AB24 3UU, UK.

***Corresponding author:** Email: p.haggarty@abdn.ac.uk

Table S1: Target regions within each DMR. Genomic location of target regions (coordinates from Human genome version 37), total extent of region analysed, number of individual CpG sites analysed and designation of imprints as maternal (M) or paternal (P) dependent upon the parental origin of methylation.

DMR	Assays	Chromosome	Start	End	CpGs Analysed	Origin
NAP1L5	3	4	89618291	89618972	37	M
ZAC1	3	6	144328448	144329712	40	M
MEST1	11	7	130130418	130133143	191	M
H19/IGF2	11	11	2019951	2024329	110	P
IGF2	1	11	2154263	2154457	12	P
KvDMR	7	11	2720230	2722180	133	M
IGDMR	4	14	101275563	101277600	28	P
SNRPN	9	15	25200013	25202009	110	M
PEG3	9	19	57349766	57352390	122	M
MCTS2	2	20	30134903	30135339	32	M
L3MBTL	4	20	42142499	42143456	56	M
NESPAS	7	20	57425615	57426925	60	M
GNASXL	3	20	57430007	57430726	51	M
				TOTAL	982	

Table S2: Primer sequences, annealing temperatures and amplicon sizes for bisulphite PCRs. Start and end genomic coordinates (based on GRCh37, release 84, version of March 2016) of each assay are also tabulated along with individual assay sizes, primer sequences (5' → 3') and annealing temperatures (Ta).

DMR	Assay	Sequence	Ta	Start	End	Size
SNRPN	1	AGGGAGTTGGGATTTTTGTATT<br>CCCCAAACTATCTCTTAAAAAAAAC	58	25200013	25200251	238
	3	GGGTTTTAAAGTTTTTTGTTTTGGAGAA<br>CTCCACCCATATCCCTTACC	58	25200318	25200546	228
	4	GGTGGTGATTGGGAGTATG<br>CTACCTACRCTCAACACCCCCTAAA	58	25200491	25200702	211
	5	GGTATTTAGGGGGTGTTGAG<br>CCTCCCAACCACTTCCTAC	58	25200673	25200886	213
	6	TTGGGAGGTAGGGGGAAG<br>CCCTCCTCTACACAACAATCATTCA	58	25200878	25201081	203
	7	GGTATTAGAAGGGGTAGTAGTATAGTT<br>ATTCTACACATCATTCCAATCTTCTTAT	58	25201082	25201302	221
	8	TTTTTGTTGGGAATTAGGTTTTGG<br>CTAAACACACACCACCTACC	58	25201370	25201567	197
	9	AGGTGGTGTGTGTTTAGTT<br>AAACCCTCRCTCCCCTACCC	58	25201550	25201761	211
	10	GGTTTAYGTAGTTTTTGTTTGGAAGGAATG<br>AATAACCCAATACAACTACCATCC	58	25201756	25202009	253
H19/IGF2	1	GGAGTATTAGGGTTAAGGGATTTAG<br>AATCCACTCATAAAAAAACCTAAAAATTT	58	2019951	2020185	234
	2	GGATTTTTAAGTTAGGAGATTAAGGTAGAT<br>TCTCCCCTTAAATCTTAAATACTATCCC	58	2020180	2020377	197
	3	ATGGGTTTATTGGGGATAGTATTTAAGA<br>AAAAAATAAATTTACCCACAAATATTCCCC	58	2020337	2020535	198
	4	GGGAATATTTGTGGGTAAATTTATTTTT<br>ACRCCACTACTACTTTATTCTCTAATCCAA	58	2020506	2020732	226
	7	GGTTGTGATGTGTGAGTTTGTA<br>AAACCCTTCTCRCAAAATCTCTAACAAACA	58	2021176	2021350	174
	8	TTTYGGGTTTTGTGTTTGTTAGAGATTTTG<br>AAAAACATCCCAAATCATCCAAACC	58	2021308	2021552	244
	10	AAGAATGGGGATATTTTATTGTTTTTTAAG<br>AACRACAATACAAACTCACACATCACAAC	58	2021844	2022018	233
	17	GGGTTGTGATGTGTGAGTTT<br>ACTTTAATAACAATAAAATATCCCCATTCT	58	2023420	2023655	235
	18	AAGAATGGGGATATTTTATTGTTATTAAAG<br>AACTCAAACCCRCCCCAACTAAAA	58	2023625	2023826	202
	19	GAGTTGTGATGTGGGAGTTT<br>AAAAACAATAAAATATCCCAATTCCTTAAA	58	2023821	2024051	230
	20	ATGTATTTTGGGGTGAATTAAATATATAGT<br>CCAAAAAAAACCCRTCTCACTACCCTAA	58	2024085	2024329	244

IGDMR	2	TTTTTTTATTTTTGGGGGTTAAGGAAT<br>AAAACCAAAAAACCTAACAAATCAAAACA	58	101275563	101275796	233
	7	TGTTTAGGTTGTAGTGTAGTGG<br>ACTCACRCCTATAATCCCAACACT	58	101276546	101276786	240
	8	GGGTTTTATYGTGTTAGTTAGGATGGTT<br>ACCCCATCCCTAATATTCAAAAATAT	58	101276692	101276924	232
	11	GTTTATTAGTTGTTYGTGGTTTATAGTTGT<br>AACCCATAAAATCACAAACCATAAC	58	101277342	101277600	258
NESPAS	1	GGTTTTAATTTTTTATTTATTTTGGGTTGT<br>ATCCCAAAAACCAACCCTTAA	58	57425615	57425793	179
	2	GGGTTGGTTTTTGGGATTTTAT<br>CAACCATTAAACAAAAATCATACCAATC	58	57425776	57426051	275
	3	ATTGGTATGATTTTTGTTTAATGGTTGA<br>AATCTACACACTTCCAAATATCAAC	58	57426024	57426202	178
	4	GTGGGTGTAGTTGGTTAAAGTTTGAAT<br>ACCCCTACATCCACCATCCATAT	58	57426139	57426311	172
	5	ATGTAGGGGTGTAAGGATGT<br>AATCATCTAACCTACAAAACCCTAA	58	57426301	57426505	204
	6	AGGGTTTTGTAGGTTAGATGAT<br>CAAAAATAAATACCCACACCACCTAAT	58	57426482	57426713	231
	7	TGTGTATATATTAAGGTTATTAGGT<br>TAATCAATCAACTCCTTTAACCCC	57	57426668	57426925	257
MCTS2	2	AGTTATAAGAGAAGTTGGAAAATGGT<br>CTATTCCTTCRACTACTAATTCCTCTCCAA	58	30134903	30135088	185
	3	ATTTTTATTGGAGAGGAATTAGTAGT<br>ACACTTTCCTTTTCATCAAACTTC	58	30135051	30135339	288
ZAC1	3	GGATTAGGATTTTAGTGAGGTTTTAGAA<br>AAATTTCTACCTTTTACTAAATATACTCCT	58	144328448	144328666	218
	4	AGGAGTATATTTAGTAAAAGGTAGAAATTT<br>AACCAAAAAAAAAACAAAACAAAAAAAAAA	58	144328636	144328917	281
	6	GGTTGAATGATAAATGGTAGATG<br>ACCTTAACTTTACCCCCAC	60	144329448	144329712	264
MEST1	2	ATTTGGGTTTTGTTTTTGAGGGTTTTATA<br>AAACCTATCATCAAAACCTCTAACAC	58	130130418	130130716	298
	3	GGGGTAATTAGATTTTTGTAGAAGTGT<br>TCCAAAAAAAACRCTACAAACCTCCCTTC	58	130130654	130130872	218
	4	GGAGGTTTGTAGYGTTTTTTTTGGATGTAG<br>ACAATACAAAAACCCAAAAACACAAAA	58	130130847	130131073	226
	5	AAGGGGGTTTTGTTTTTTTAATTGTG<br>AAACTCTATTAAACCCACCACCAAACTAAT	60	130131093	130131346	253
	6	AGTTTGGTGGTGGGTTTAATAGAGT<br>CCCCTAAATACCCCAACTCTTTCCTT	58	130131319	130131618	299
	7	GGTGGTTTAAGGAAAGAGTTGG<br>CCACAAAAATAAAATACCCCTCTAACAAT	58	130131584	130131791	207
	8	AGAGGGGTATTTTATTTTTGTGGTTA<br>CAACCCRCAACCCACAACCC	58	130131768	130132062	294
	9	GGGTTGTGGGTTGYGGGTTG<br>AATTTTCRCCCAAAAACAACCCCAACTC	58	130132042	130132294	252
	10	GGAAYGAGGGGGTGTGGTTG<br>AAAATCCAAAAATCCTAAATCTCACC	58	130132195	130132450	256

	12	GTTTTGGTTTGATTTAGGATATTTAGATTT<br>ATACCTAAATCTTAAAATCCTAAACTACAC	58	130132607	130132917	310
	13	GGTGTAGTTTAGGATTTTAAGATTTAGG<br>CACACTCCCRTAATAATACCACCCAACCTA	58	130132886	130133143	257
KvDMR	2	GGGTTTTGATTTAGAATTATTAAGTGGATT<br>AAACAATACRCTCCCATCTACACCTTATAA	58	2720230	2720499	269
	3	TTGGTTAYGTTGTTTATAAGGTGTAGATGG<br>ATTCTCCCAAACTCTCCTCAA	58	2720456	2720672	216
	4	TTAAATTTTTTTAGAGAGATGGGGAGGG<br>CTATTCTCCCCRAAAACTCCTCAACATA	58	2720618	2720822	204
	6	TTGAGGAGTTTTTTGGAGGTT<br>CACACCCAACCAATACCTCATA	58	2721176	2721465	288
	7	GTATTGGTTGGGTGTGAGG<br>CCTAAACRCCCACAAACCTCCAC	58	2721448	2721689	241
	8	GTGGGTTTTYGGTGTGGAGGT<br>CATATCCCYACCACCCCTACCACTC	58	2721653	2721924	272
	9	AGGGAAGTTTTAGGGTGTGAATTTTTAGAG<br>CCAAACCACCCACCTAACAAAAAAC	60	2721925	2722180	255
PEG3	7	GAGGGGTAGTGGAGGTGA<br>CTCACCCCTTCCCCTATTATT	58	57349766	57349970	204
	8	GGTGAATAAAGTTTTGGTTAGGTAATAAT<br>ACTATTTTACCAATAAAACTAACTCAATCC	58	57349926	57350129	203
	9	GTTTTTTTAGATGYGGGGATTGAGTTAGT<br>CCCCRCCCACCTAAATACCATCTTTAA	58	57350083	57350297	214
	10	TTAGGTGGGYGGGGTTTGAATAGAT<br>TTATTCAAACCCCACCCACCTAAAC	58	57350283	57350529	246
	12	GGGYGGGGTTTGAGTGGATAGTTATTTAA<br>CCCCACCCACTAAATACCATCTTTTATA	58	57350926	57351197	271
	13	TTAGTGGGTGGGGTTTGAGTA<br>AATCTAAAAACTACRAAAATACCCCTTCCT	58	57351184	57351484	300
	14	GTGGTAGAGTTTTYGGTTGTTTGTTTTAAT<br>CTTACCRCAAACCCAAAACCCC	58	57351569	57351869	300
	16	GTGTAGAAGTTTGGGTAGTTG<br>CTCACCTCACCTCAATACTAC	54	57351943	57352095	152
	18	AGGYGGGGTTTTTGTGTTAGTTAAT<br>ACATCTCAATAAAATTTCCTCCATT	58	57352139	57352390	251
L3MBTL	2	TGTTTAGTAGTGTTTTGGATGTGTT<br>ACTTAACCAATCCCCACAAACA	58	42142499	42142729	230
	3	GTTTGTGGGGATTGGTTAAGT<br>CAAACTAACACAAACCCACTCAAAATAC	58	42142708	42142991	283
	4	GGGGTATTTTGAGTGGGTTTGTGTTA<br>ACCCTCTCTTCRCCTCATACCAACTCAC	58	42142960	42143203	243
	5	TATGAGGYGAAGAGAGGGTTATGGT<br>ACAAAACCCAACTCAAAACCT	58	42143184	42143456	272
GNASXL	7	GTGTATTAYGATGAAGGGGTGGTTAGTA<br>CTTCTCCRCCAAAAATACCTTCTTAACCTT	58	57430007	57430254	247
	8	GTTTTTTTAAGGTTAAGAAGGTATTTTTGG<br>TAACTAAACCCCCTCCTCCCA	58	57430216	57430504	288
	9	GAGGAGGGGGTTTAGTTAAAGG<br>CCCTCCCCRACAATTCCTAACTCAC	58	57430486	57430726	240

NAP1L5	2	ATTTTGGGAAAAATAAAATTAGGTTTTTTT<br>AAAATATAATAACATCTATAAACCCCTACT	58	89618291	89618586	295
	3	TTTGGATTTTGGYGAGTAGGGGTTTATAGA<br>CCAAACCCCTACAAAAAATACCCC	58	89618542	89618742	200
	4	TTTTTYGGTTTTGGGGTATTTTTTGTAGGG<br>CTAAACCAACTCRAATACAAAACCTCTAC	58	89618706	89618972	266
IGF2	1	GGGTTTTGGGTGGGTAGAGT<br>CCAAAACAACTTCCCCAAAT	60	2154263	2154457	194

Table S3: Mean methylation across each DMR. Mean methylation and standard deviation were calculated across all CpG sites within each DMR. Methylation values were included in mean calculations only when cover on each strand was at least 250x, and methylation values less than 10% or greater than 90% were excluded. Only individuals with sex, total intracranial volume and age data were included.

DMR	n	Methylation	sd
NAP1L5	223	62.3	4.2
ZAC1	184	54.4	3.7
MEST1	233	53.8	1.7
H19/IGF2	233	55.6	1.8
IGF2	227	56.3	5.9
KvDMR	230	54.9	2.7
IGDMR	204	71.9	4.4
SNRPN	234	50.0	1.7
PEG3	217	62.2	5.1
MCTS2	212	57.5	5.6
L3MBTL	225	48.6	4.1
NESPAS	234	57.2	2.8
GNASXL	232	53.4	2.6

Figure S1; coefficient plot for associations between imprint methylation and hippocampal volumes and hyperintensities on chromosomes 6 (*ZAC1*) and 7 (*MEST1*) in ABC36 participants at baseline image collection. The schematic at the top of each column of figures shows the genomic location of the individual CpG sites in the context of function on the maternally and paternally inherited alleles (indicated by male and female symbols). The y axes represent hippocampal volume (in mm^3) or number of lesions per percent methylation and the x axes genomic location on chromosome 6 or 7, respectively (GRCh37, release 84, version of March 2016). Positions significant at $p<0.05$ are shown in blue when coefficients are negative and in red when positive. Regressions were adjusted for sex, total intracranial volume and age at baseline MRI (in days). Created using STATA/MP version 15 (StataCorp. 2017. *Stata Statistical Software: Release 15*. College Station, TX: StataCorp LLC).

Figure S2; coefficient plot for associations between imprint methylation and hippocampal volumes and hyperintensities on chromosomes 15 (*SNRPN*) and 19 (*PEG3*) in ABC36 participants at baseline image collection. The schematic at the top of each column of figures shows the genomic location of the individual CpG sites in the context of function on the maternally and paternally inherited alleles (indicated by male and female symbols). The y axes represent hippocampal volume (in mm^3) or number of lesions per percent methylation and the x axes genomic location on chromosome 15 or 19, respectively (GRCh37, release 84, version of March 2016). Positions significant at $p<0.05$ are shown in blue when coefficients are negative and in red when positive. Regressions were adjusted for sex, total intracranial volume and age at baseline MRI (in

days). Created using STATA/MP version 15 (StataCorp. 2017. *Stata Statistical Software: Release 15*. College Station, TX: StataCorp LLC).

Figure S3; coefficient plot for associations between imprint methylation and hippocampal volumes and hyperintensities on chromosome 11 (*H19/IGF2*, *IGF2*, *KvDMR*) in ABC36 participants at baseline image collection. The schematic at the top of each column of figures shows the genomic location of the individual CpG sites in the context of function on the maternally and paternally inherited alleles (indicated by male and female symbols). The y axes represent hippocampal volume (in mm^3) or number of lesions per percent methylation and the x axes genomic location on chromosome 11 (GRCh37, release 84, version of March 2016). Positions significant at $p<0.05$ are shown in blue when coefficients are negative and in red when positive. Regressions were adjusted for sex, total intracranial volume and age at baseline MRI (in days). Created using STATA/MP version 15 (StataCorp. 2017. *Stata Statistical Software: Release 15*. College Station, TX: StataCorp LLC).

Figure S4; coefficient plot for associations between imprint methylation and hippocampal volumes and hyperintensities on chromosome 20 (*MCTS2*, *L3MBTL*, *NESPAS*, *GNASXL*) in ABC36 participants at baseline image collection. The schematic at the top of each column of figures shows the genomic location of the individual CpG sites in the context of function on the maternally and paternally inherited alleles (indicated by male and female symbols). The y axes represent hippocampal volume (in mm^3) or number of lesions per percent methylation and the x axes genomic location on

chromosome 11 (GRCh37, release 84, version of March 2016). Positions significant at $p<0.05$ are shown in blue when coefficients are negative and in red when positive. Regressions were adjusted for sex, total intracranial volume and age at baseline MRI (in days). Created using STATA/MP version 15 (StataCorp. 2017. *Stata Statistical Software: Release 15*. College Station, TX: StataCorp LLC).

Figure S5; coefficient plot for associations between imprint methylation and hippocampal volumes and hyperintensities on chromosomes 4 (*NAP1L5*) and 14 (*IGDMR*) in ABC36 participants at baseline image collection. The schematic at the top of each column of figures shows the genomic location of the individual CpG sites in the context of function on the maternally and paternally inherited alleles (indicated by male and female symbols). The y axes represent hippocampal volume (in mm^3) or number of lesions per percent methylation and the x axes genomic location on chromosome 15 or 19, respectively (GRCh37, release 84, version of March 2016). Positions significant at $p<0.05$ are shown in blue when coefficients are negative and in red when positive. Regressions were adjusted for sex, total intracranial volume and age at baseline MRI (in days). Created using STATA/MP version 15 (StataCorp. 2017. *Stata Statistical Software: Release 15*. College Station, TX: StataCorp LLC).

Figure S6; coefficient plot for associations between imprint methylation and change in hippocampal volumes and hyperintensities on chromosomes 6 (*ZAC1*) and 7 (*MEST1*) in ABC36 participants between baseline and follow-up image collection. The schematic at the top of each column of figures shows the genomic location of the individual CpG

sites in the context of function on the maternally and paternally inherited alleles (indicated by male and female symbols). The y axes represent change in hippocampal volume (in mm^3) or the change in number of lesions per percent methylation and the x axes genomic location on chromosome 6 or 7, respectively (GRCh37, release 84, version of March 2016). Positions significant at $p<0.05$ are shown in blue when coefficients are negative and in red when positive. Regressions were adjusted for sex, total intracranial volume, age at baseline MRI (in days) and time between MRI scans (in days). Created using STATA/MP version 15 (StataCorp. 2017. *Stata Statistical Software: Release 15*. College Station, TX: StataCorp LLC).

Figure S7; coefficient plot for associations between imprint methylation and change in hippocampal volumes and hyperintensities on chromosomes 15 (*SNRPN*) and 19 (*PEG3*) in ABC36 participants between baseline and follow-up image collection. The schematic at the top of each column of figures shows the genomic location of the individual CpG sites in the context of function on the maternally and paternally inherited alleles (indicated by male and female symbols). The y axes represent change in hippocampal volume (in mm^3) or the change in number of lesions per percent methylation and the x axes genomic location on chromosome 15 or 19, respectively (GRCh37, release 84, version of March 2016). Positions significant at $p<0.05$ are shown in blue when coefficients are negative and in red when positive. Regressions were adjusted for sex, total intracranial volume, age at baseline (in days) and time between MRI scans (in days). Created using STATA/MP version 15 (StataCorp. 2017. *Stata Statistical Software: Release 15*. College Station, TX: StataCorp LLC).

Figure S8; coefficient plot for associations between imprint methylation and change in hippocampal volumes and hyperintensities on chromosome 11 (*H19/IGF2*, *IGF2*, *KvDMR*) in ABC36 participants between baseline and follow-up image collection. The schematic at the top of each column of figures shows the genomic location of the individual CpG sites in the context of function on the maternally and paternally inherited alleles (indicated by male and female symbols). The y axes represent change in hippocampal volume (in mm^3) or the change in number of lesions per percent methylation and the x axes genomic location on chromosome 11 (GRCh37, release 84, version of March 2016). Positions significant at $p<0.05$ are shown in blue when coefficients are negative and in red when positive. Regressions were adjusted for sex, total intracranial volume, age at baseline (in days) and time between MRI scans (in days). Created using STATA/MP version 15 (StataCorp. 2017. *Stata Statistical Software: Release 15*. College Station, TX: StataCorp LLC).

Figure S9; coefficient plot for associations between imprint methylation and change in hippocampal volumes and hyperintensities on chromosomes 4 (*NAP1L5*) and 14 (*IGDMR*) in ABC36 participants between baseline and follow-up image collection. The schematic at the top of each column of figures shows the genomic location of the individual CpG sites in the context of function on the maternally and paternally inherited alleles (indicated by male and female symbols). The y axes represent change in hippocampal volume (in mm^3) or the change in number of lesions per percent methylation and the x axes genomic location on chromosome 4 or 14, respectively (GRCh37, release 84, version of March 2016). Positions significant at $p<0.05$ are

shown in blue when coefficients are negative and in red when positive. Regressions were adjusted for sex, total intracranial volume, age at baseline (in days) and time between MRI scans (in days). Created using STATA/MP version 15 (StataCorp. 2017. *Stata Statistical Software: Release 15*. College Station, TX: StataCorp LLC).

Figure S1

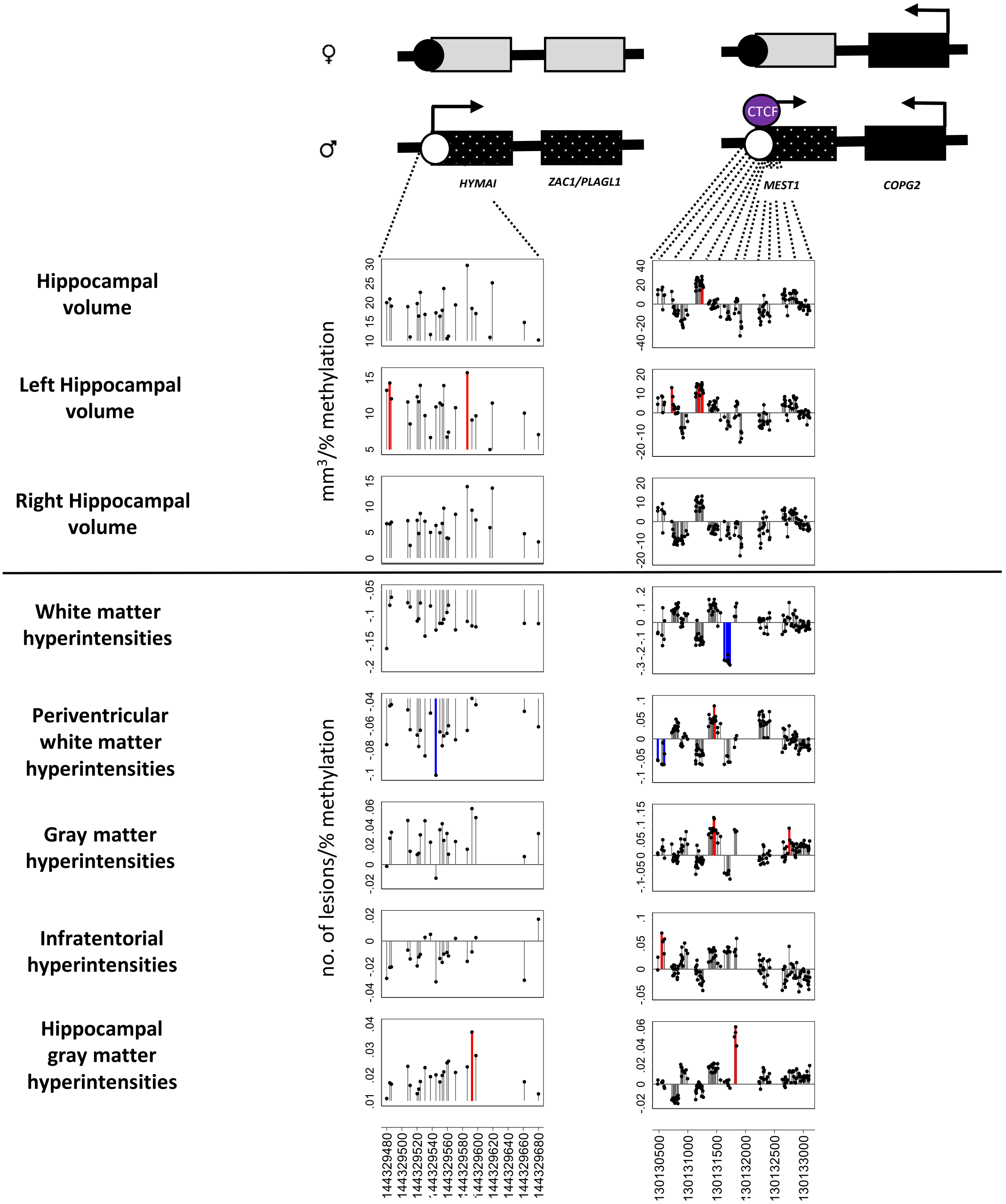

♀
♂
CTCF
HYMAI
ZAC1/PLAGL1
MEST1
COPG2
Hippocampal volume
Left Hippocampal volume
Right Hippocampal volume
White matter hyperintensities
Periventricular white matter hyperintensities
Gray matter hyperintensities
Infratentorial hyperintensities
Hippocampal gray matter hyperintensities
mm3/% methylation
no. of lesions/% methylation

Figure S2

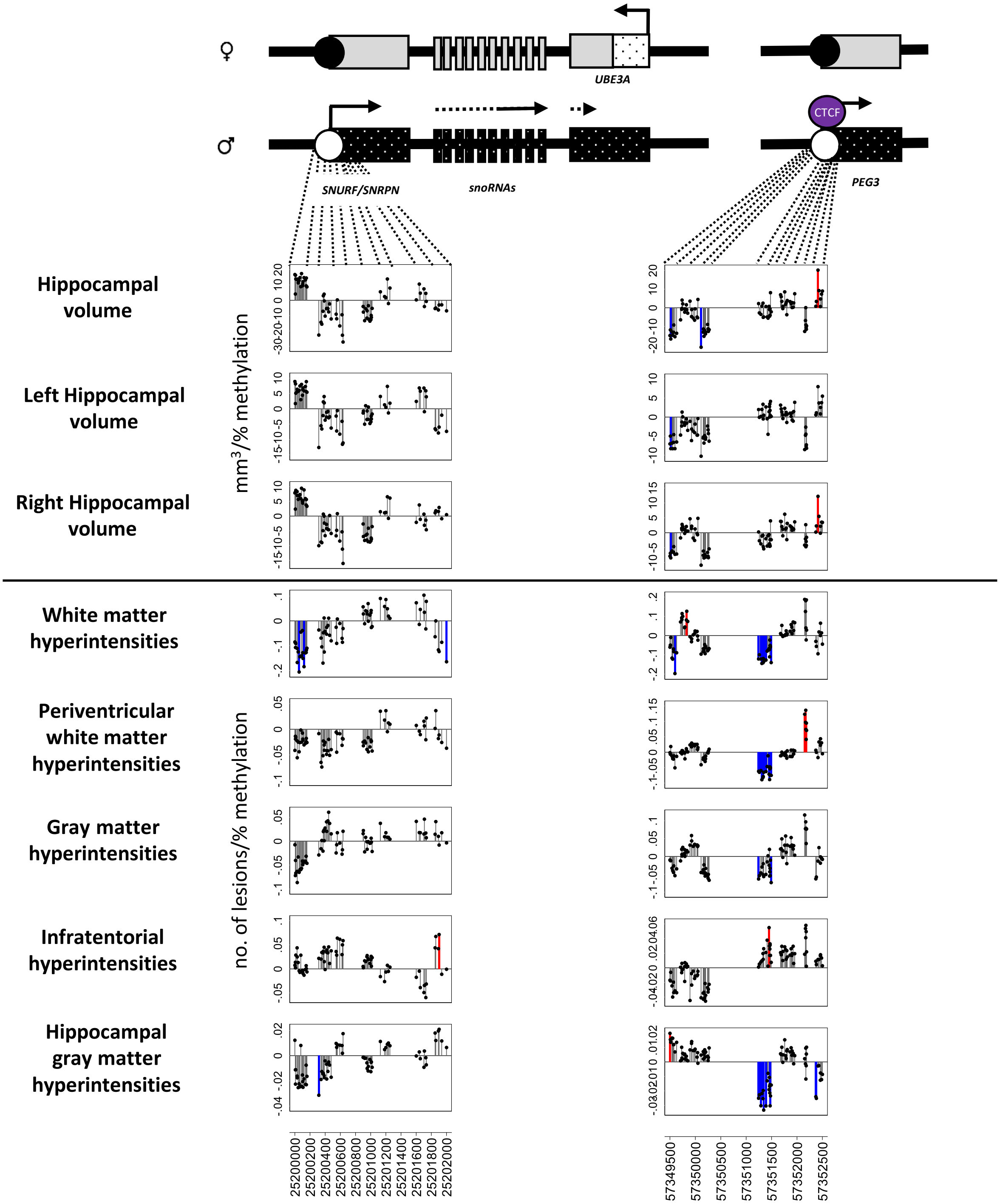

♀
♂
UBE3A
CTCF
SNURF/SNRPN
snoRNAs
PEG3
Hippocampal volume
Left Hippocampal volume
Right Hippocampal volume
mm3/% methylation
White matter hyperintensities
Periventricular white matter hyperintensities
Gray matter hyperintensities
Infratentorial hyperintensities
Hippocampal gray matter hyperintensities
no. of lesions/% methylation
25200000
25200200
25200400
25200600
25200800
25201000
25201200
25201400
25201600
25201800
25202000
57349500
57350000
57350500
57351000
57351500
57352000
57352500

Figure S3

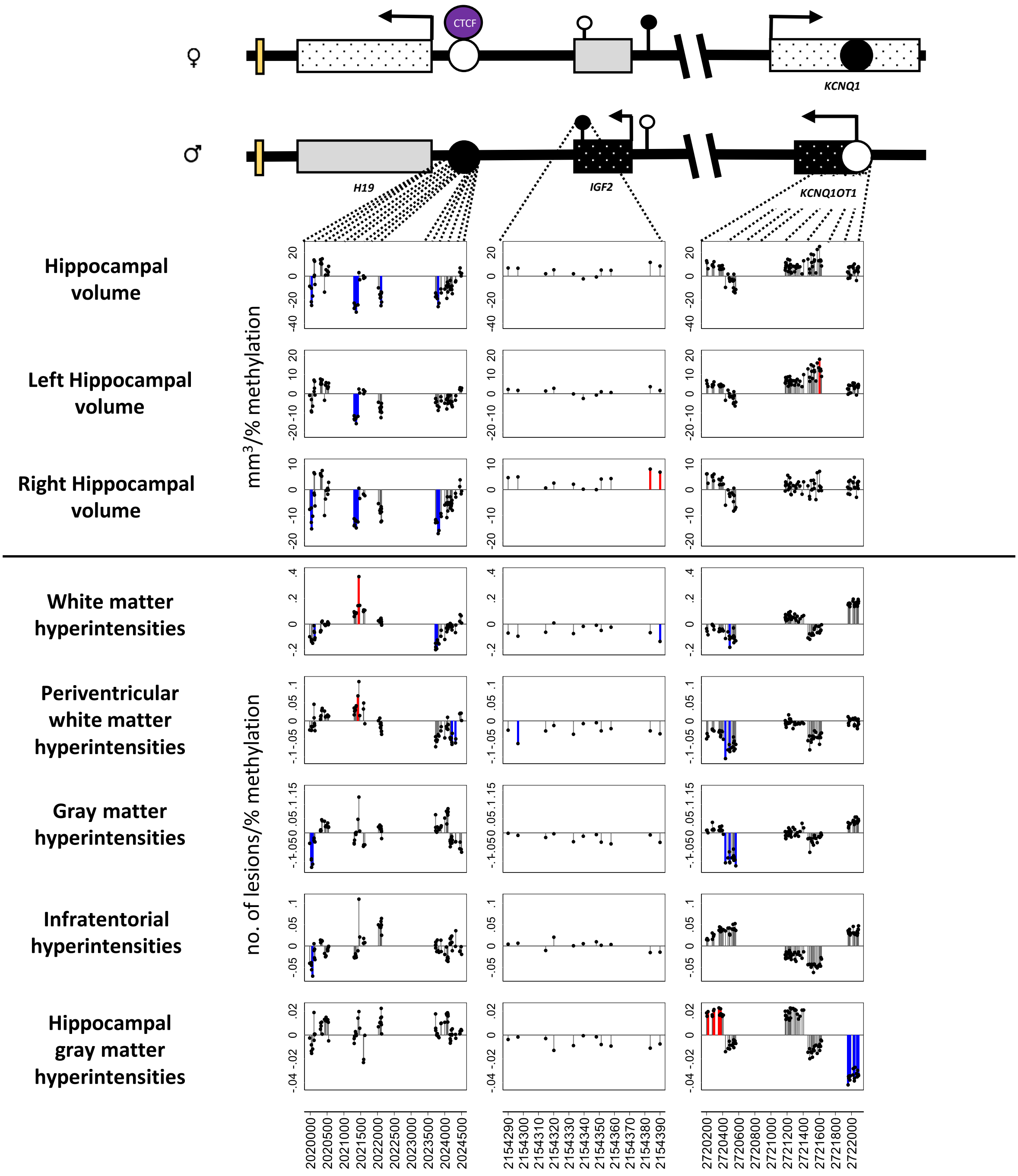

CTCF
KCNQ1
H19
IGF2
KCNQ1OT1
Hippocampal volume
Left Hippocampal volume
Right Hippocampal volume
mm3/% methylation
White matter hyperintensities
Periventricular white matter hyperintensities
Gray matter hyperintensities
Infratentorial hyperintensities
Hippocampal gray matter hyperintensities
no. of lesions/% methylation

Figure S4

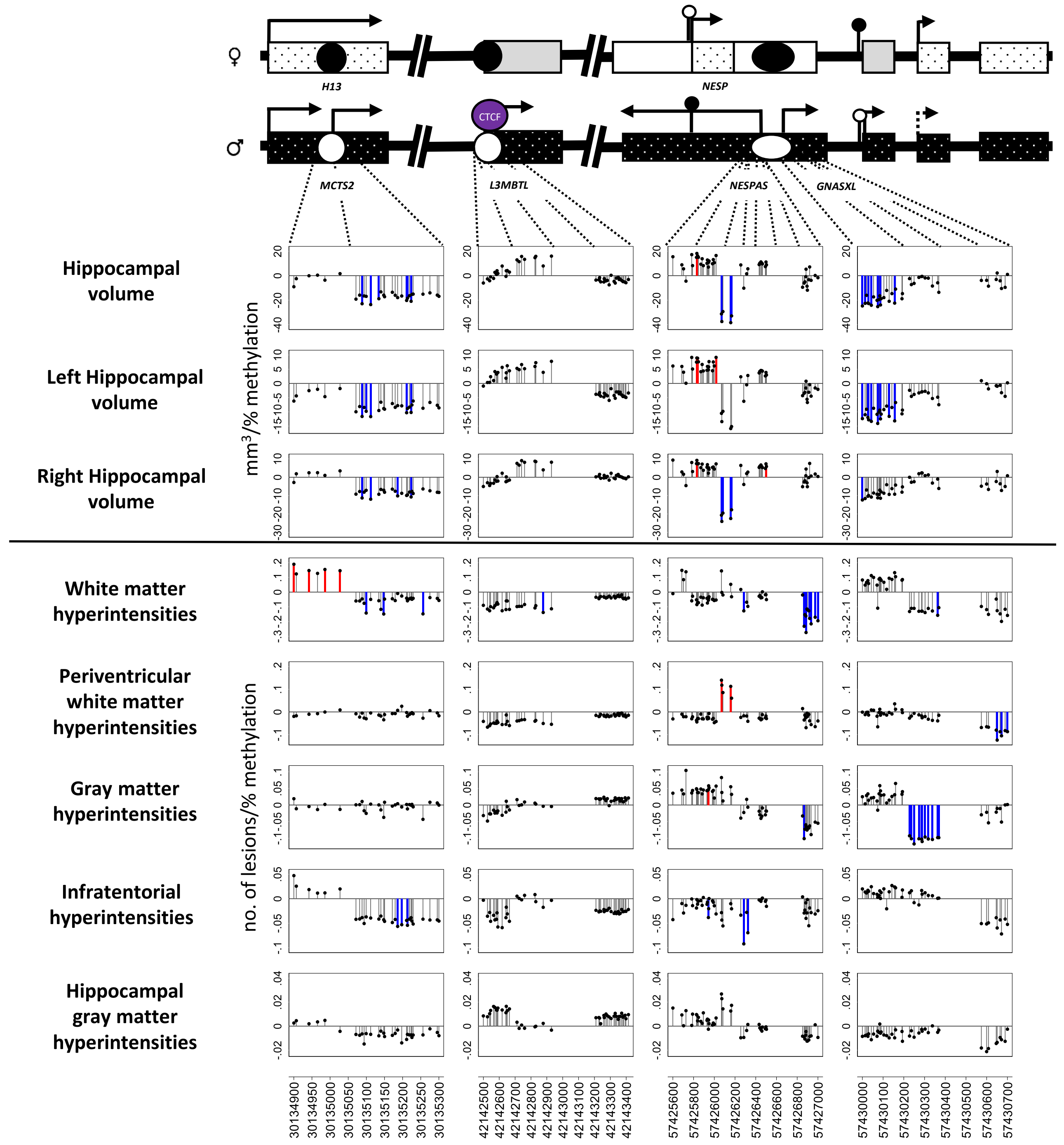

♀
H13
NESP
♂
CTCF
MCTS2
L3MBTL
NESPAS
GNASXL
Hippocampal volume
Left Hippocampal volume
Right Hippocampal volume
mm³/% methylation
White matter hyperintensities
Periventricular white matter hyperintensities
Gray matter hyperintensities
Infratentorial hyperintensities
Hippocampal gray matter hyperintensities
no. of lesions/% methylation

Figure S5

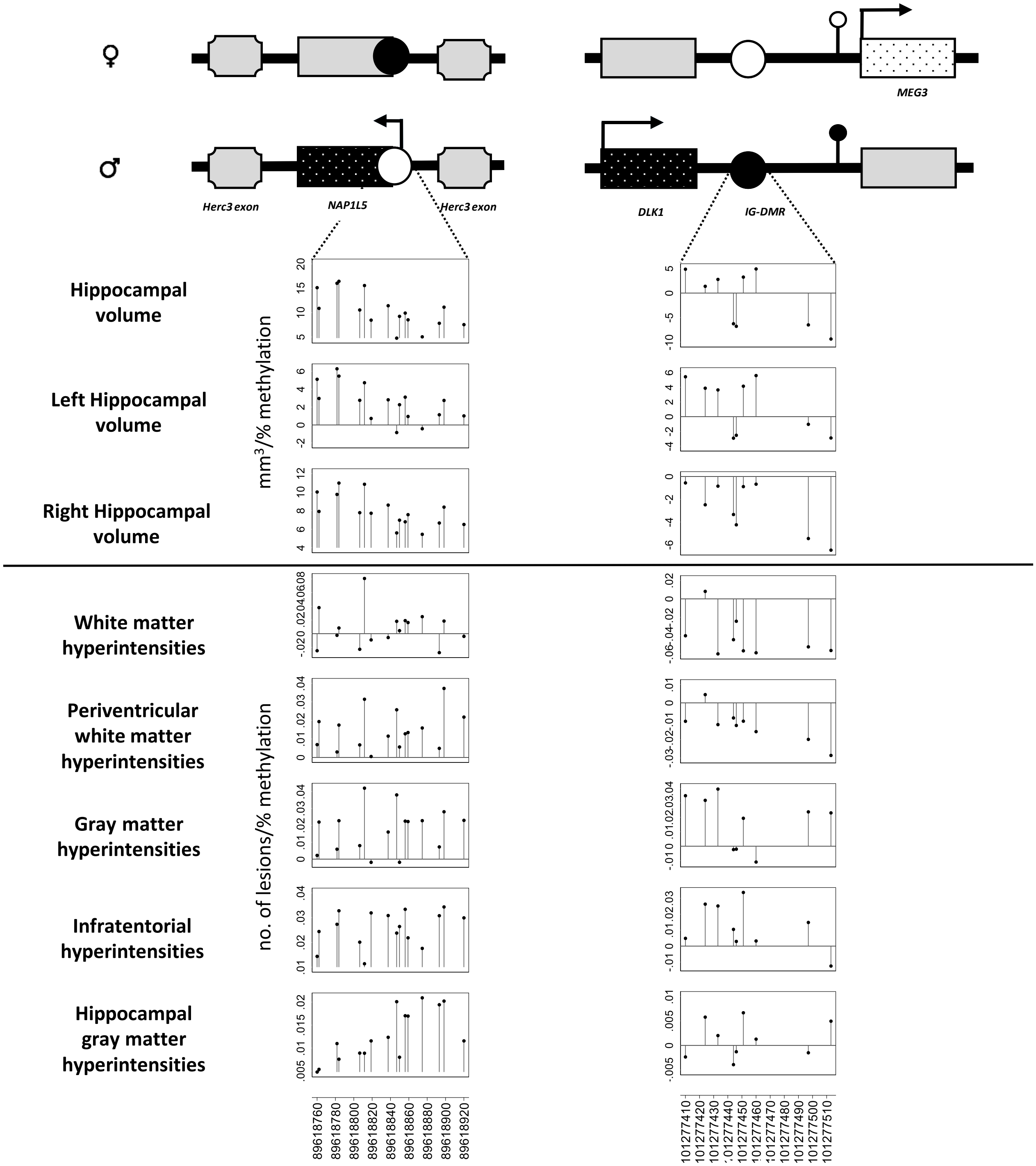

♀
♂
Herc3 exon
NAP1L5
Herc3 exon
DLK1
IG-DMR
MEG3
Hippocampal volume
Left Hippocampal volume
Right Hippocampal volume
mm3/% methylation
White matter hyperintensities
Periventricular white matter hyperintensities
Gray matter hyperintensities
Infratentorial hyperintensities
Hippocampal gray matter hyperintensities
no. of lesions/% methylation

Figure S6

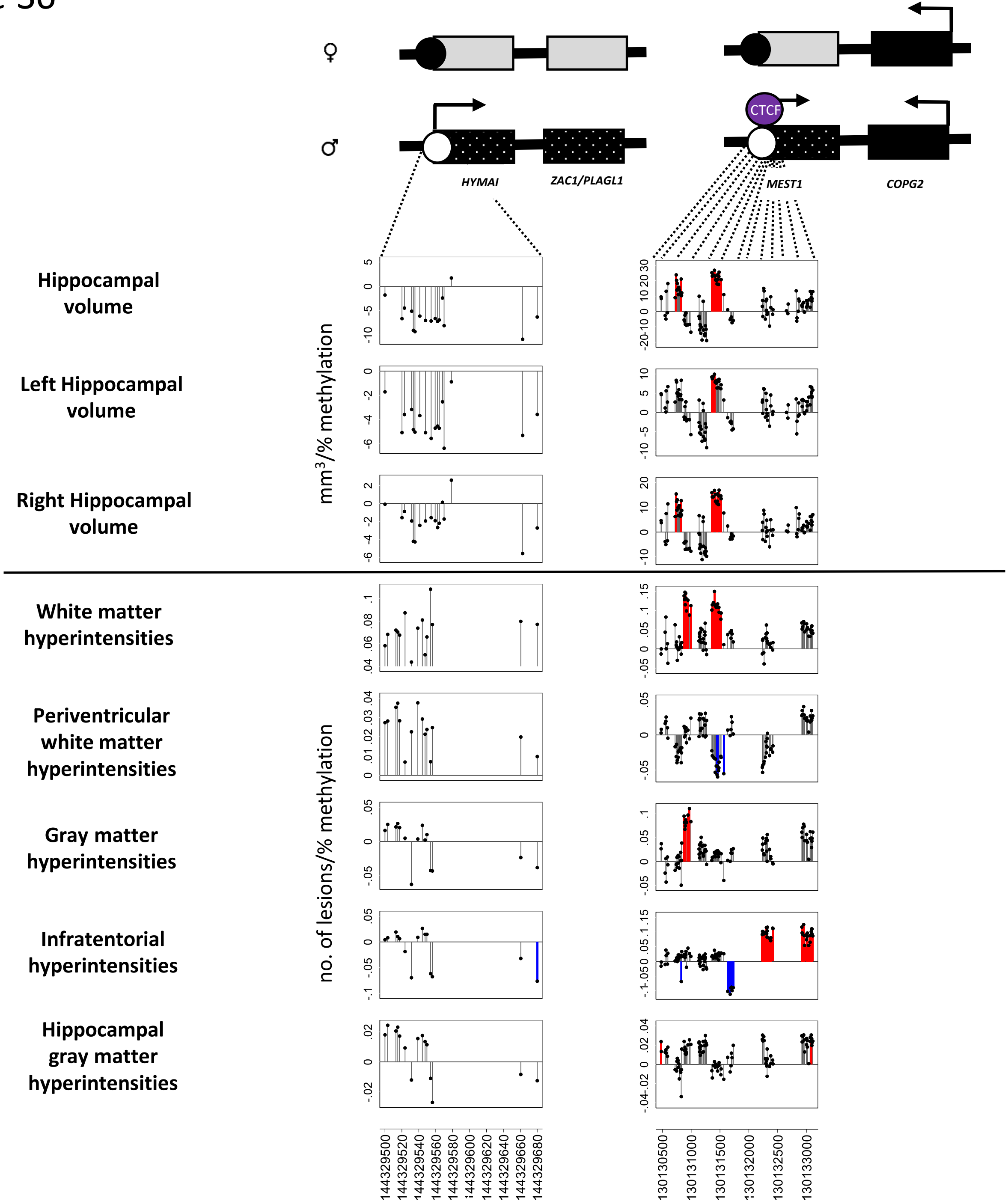

♀
♂
CTCF
HYMAI
ZAC1/PLAGL1
MEST1
COPG2
Hippocampal volume
Left Hippocampal volume
Right Hippocampal volume
White matter hyperintensities
Periventricular white matter hyperintensities
Gray matter hyperintensities
Infratentorial hyperintensities
Hippocampal gray matter hyperintensities
mm³/% methylation
no. of lesions/% methylation

Figure S7

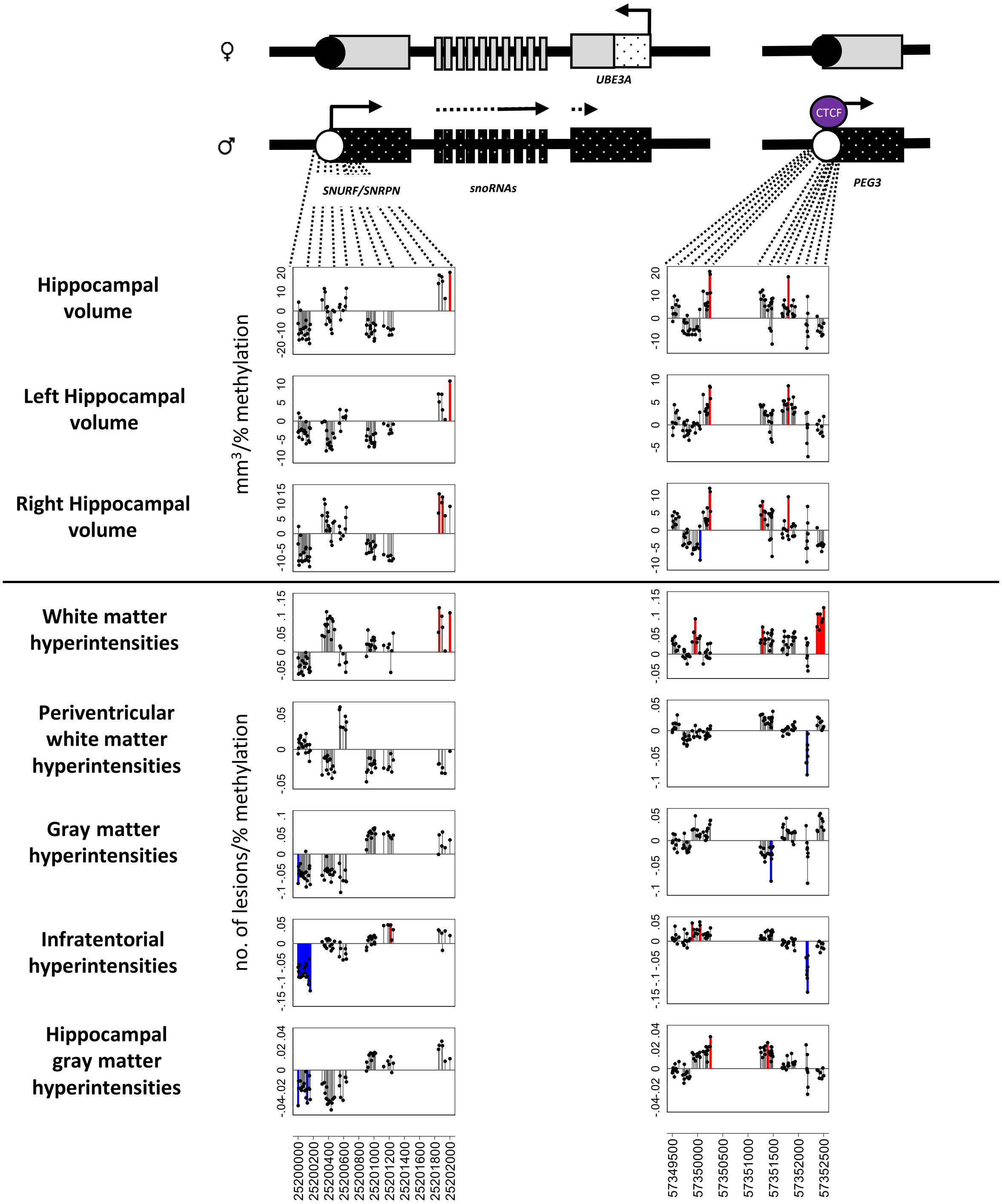

♀
♂
UBE3A
CTCF
SNURF/SNRPN
snoRNAs
PEG3
Hippocampal volume
Left Hippocampal volume
Right Hippocampal volume
mm3/% methylation
White matter hyperintensities
Periventricular white matter hyperintensities
Gray matter hyperintensities
Infratentorial hyperintensities
Hippocampal gray matter hyperintensities
no. of lesions/% methylation
25200000
25200200
25200400
25200600
25200800
25201000
25201200
25201400
25201600
25201800
25202000
57349500
57350000
57350500
57351000
57351500
57352000
57352500

Figure S8

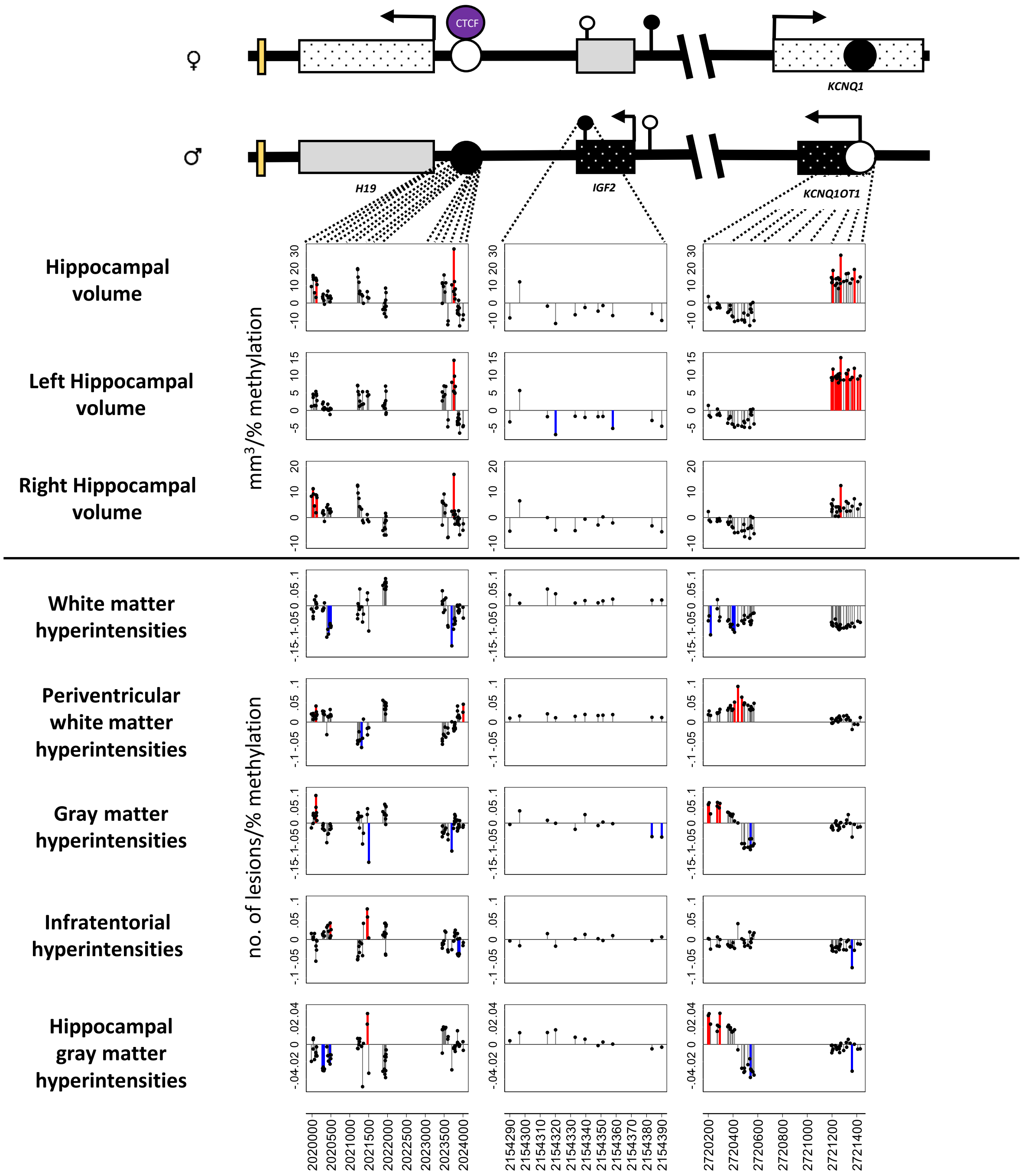

CTCF
♀
♂
KCNQ1
H19
IGF2
KCNQ1OT1
Hippocampal volume
Left Hippocampal volume
Right Hippocampal volume
mm3/% methylation
White matter hyperintensities
Periventricular white matter hyperintensities
Gray matter hyperintensities
Infratentorial hyperintensities
Hippocampal gray matter hyperintensities
no. of lesions/% methylation

Figure S9

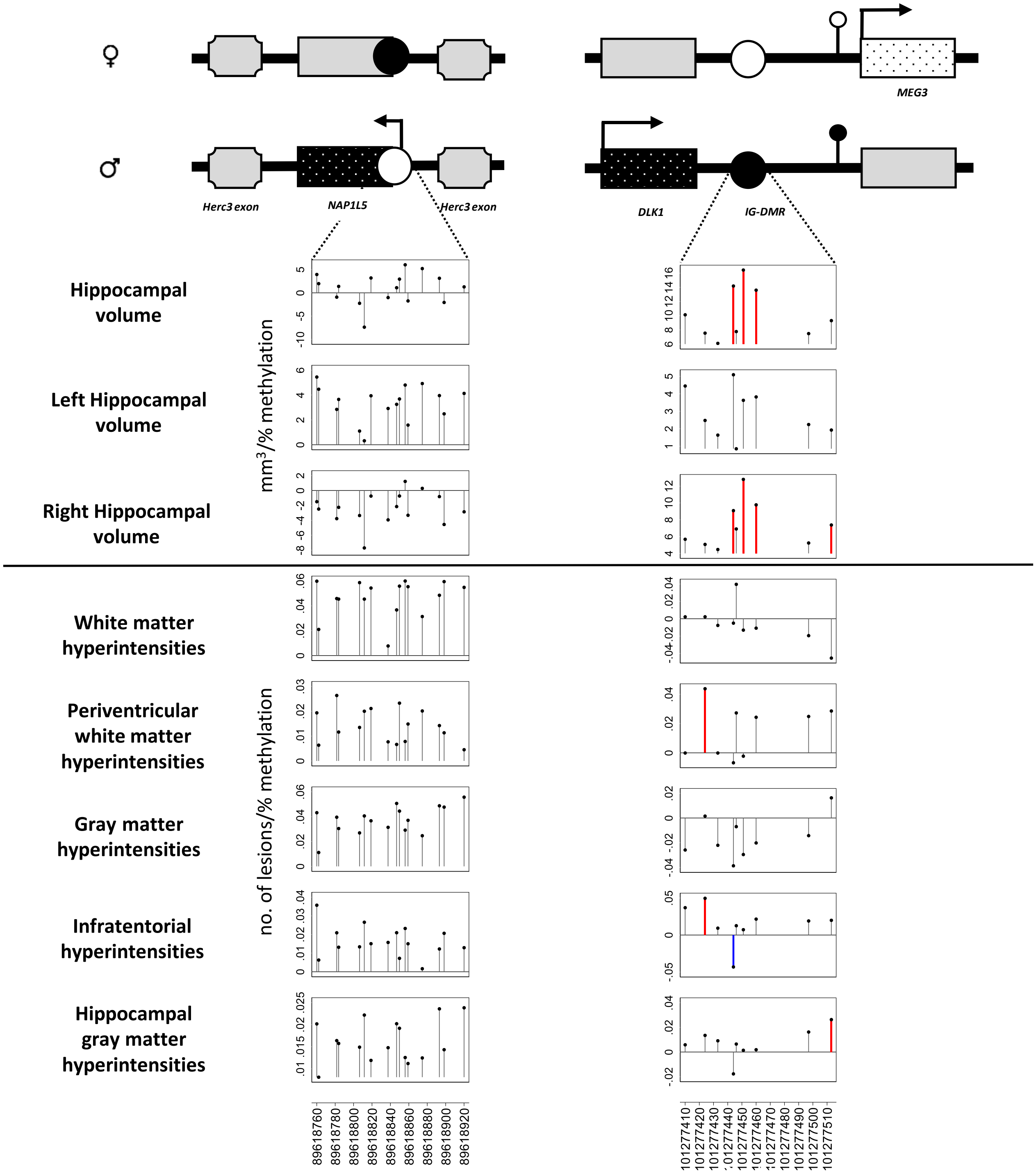

♀
♂
MEG3
Herc3 exon
NAP1L5
Herc3 exon
DLK1
IG-DMR
Hippocampal volume
Left Hippocampal volume
Right Hippocampal volume
mm3/% methylation
White matter hyperintensities
Periventricular white matter hyperintensities
Gray matter hyperintensities
Infratentorial hyperintensities
Hippocampal gray matter hyperintensities
no. of lesions/% methylation
89618760
89618780
89618800
89618820
89618840
89618860
89618880
89618900
89618920
101277410
101277420
101277430
101277440
101277450
101277460
101277470
101277480
101277490
101277500
101277510
